# Heart Regeneration and Repair: Molecular Mechanism and Therapeutic Targets

**DOI:** 10.1002/mco2.70407

**Published:** 2025-10-04

**Authors:** Mingchuan Liu, Tingwei Peng, Rui Yu, Kexin Wang, Di Wang, Xiaojie Jia, Yan Zhang, Jianqiang Hu, Bingchao Qi, Yan Li

**Affiliations:** ^1^ Department of Cardiology Tangdu Hospital Fourth Military Medical University Xi'an China

**Keywords:** cardiomyocyte proliferation, heart regeneration, metabolic reprogramming, myocardial infarction, therapeutics

## Abstract

The substantial loss of cardiomyocytes resulting from myocardial infarction leads to pathological remodeling of the heart and the onset of heart failure. Promoting heart regeneration is therefore a critical therapeutic goal for repairing damaged cardiac tissue. Over the past two decades, the utilization of cardiac stem cells for heart regeneration has emerged as a focal point of research. However, the related mechanisms and efficacy remain constrained by poor integration and survival. Concurrently, genetic lineage tracing has definitively shown that the adult mammalian heart lacks significant endogenous stem cells. It is now widely accepted that heart regeneration primarily arises from the proliferation of pre‐existing adult cardiomyocytes. This review systematically summarizes the physiological and microenvironmental changes during the developmental process of cardiomyocytes, elucidates the intrinsic and extrinsic molecular biological mechanisms that regulate cardiomyocyte proliferation, and discusses exogenous cell transplantation therapy, potentially endogenous pharmacological and genetic approaches, as well as promising bioengineering and cross‐disciplinary methods. By synthesizing these multifaceted advances, this review aims to clarify important issues that require further elucidation in this field, thereby advancing the depth of research on heart regeneration and its clinical translational applications.

## Graphical abstract

Regarding the general picture of heart regeneration therapy, it encompasses exogenous cell therapy, endogenous medications, metabolism, interventions in gene pathways, as well as the therapeutic strategies in the burgeoning interdisciplinary field of medicine and engineering in recent years.

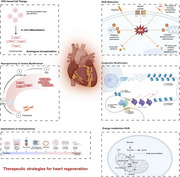

## 1 Introduction

Heart failure is a severe manifestation or advanced stage of various heart diseases. The prevalence of heart failure ranges from 1.0 to 2.0%, with persistently high mortality and readmission rates [1, 2, 3]. Particularly in China, the age‐standardized prevalence of heart failure is 1032.84 per 100,000 people ranking first among all Asian countries [4]. Ischemic cardiomyopathy is one of the main causes of heart failure, accounting for 37.32%. Although breakthroughs have been made in recent research on anti‐heart failure, lipid lowering, and anticoagulant drugs, as well as cardiac implantable devices, the proliferative capacity of adult mammalian cardiomyocytes is very limited. Current treatment methods still cannot reverse the massive loss of cardiomyocytes after myocardial damage [5, 6]. Heart transplantation is currently the only radical treatment for severe heart failure. However, due to the shortage of donors, the extremely limited number of transplantations is a drop in the bucket for the large population of heart failure patients. Therefore, the long‐term prognosis of heart failure after myocardial infarction (MI) remains poor. If the regeneration and repair of the myocardium can be initiated to replace the infarcted myocardium, it is expected to fundamentally cure MI and other heart diseases, which is currently a research frontier and hotspot in the field of heart failure.

Previous perspectives held that the cardiomyocytes of common mammals are predominantly polyploid after birth, with humans exhibiting over 90% polyploidy, resulting in extremely low differentiation and regenerative capacity (<1%), which is only sufficient to maintain routine cardiomyocyte turnover. The heart exhibits minimal regenerative potential, as its cellular composition lacks a progenitor cell population, and cardiomyocytes exit the cell cycle shortly after birth. Consequently, even following cardiac injury, these cells do not re‐enter the proliferative cycle. Thus, any loss of cardiomyocytes is essentially irreversible and may contribute to or exacerbate heart failure. However, cardiac regeneration is widely observed in amphibians and fish [7], and mammals such as mice retain this capacity during developmental and neonatal stages [8]. Additionally, organ regeneration occurs in adult mammals, such as liver regeneration [9]. Therefore, identifying methods to promote cardiac regeneration in adult mammals has been a persistent goal in cardiovascular research. Over the past two decades, fundamental and clinical research on heart regeneration has primarily focused on cell‐based therapies, including bone marrow‐derived cells, mesenchymal stem cells (MSCs), and endogenous cardiac stem cells. Among these, the most notable and influential studies were those initiated by the Anversa team in 2001, investigating c‐Kit^+^ bone marrow stem cells and cardiac stem cells [10, 11]. However, despite the established safety of these clinical trials over the years, their efficacy in improving cardiac function has been minimal. Further studies revealed that these cells do not directly differentiate into cardiomyocytes but instead exert beneficial effects primarily through paracrine mechanisms, promoting angiogenesis and inhibiting cardiomyocyte death [12]. Based on this understanding, researchers attempted to differentiate pluripotent stem cells including embryonic stem cells (ESCs) and induced pluripotent stem cells (iPSCs) into cardiomyocytes in vitro before transplanting them into damaged hearts. However, experimental results showed poor long‐term engraftment and a risk of arrhythmogenesis [13]. Consequently, identifying methods to stimulate the proliferation of endogenous adult cardiomyocytes for cardiac regeneration has emerged as a critical research direction. Given that adult zebrafish and neonatal mice primarily rely on cardiomyocyte proliferation to sustain cardiac regenerative capacity [14, 15] and that adult humans and mice exhibit annual cardiomyocyte turnover—albeit at a low rate [16], studies have confirmed that cardiomyocyte renewal following apical resection in adult mice and post‐MI in large animals such as pigs originates from the proliferation of resident cardiomyocytes [17, 18]. This suggests that adult cardiomyocytes retain intrinsic proliferative potential, which has spurred global research into the regulation of endogenous cardiomyocyte regeneration, namely, stimulating pre‐existing cardiomyocytes to proliferate, generate new cardiomyocytes, and acquire mature functional properties to replenish lost cells.

After nearly 20 years of exploration, it has been widely recognized that heart regeneration mainly stems from the proliferation of pre‐existing cardiomyocytes [19, 20, 21]. In recent years, the rapid development of research in this field has enabled people to gain a deeper understanding of heart regeneration. Knowledge regarding the characteristics of endogenous cells and microenvironments, exogenous influencing factors, as well as the molecular biological mechanisms and intervention strategies of myocardial cell regeneration is constantly being updated and enriched. Meanwhile, considering that the current literature summaries in this field are still not comprehensive and systematic enough. Therefore, we believe it is necessary to conduct a detailed and thorough review of the relevant physiological characteristics, regulatory mechanisms, and intervention strategies of mammalian myocardial cell regeneration, and to clarify the important issues that still need further elucidation and potential solutions, so as to promote in depth research and translational application in this field.

Herein, in our review, we first comprehensively discussed the physiological and microenvironmental changes of cardiomyocytes during development, emphasizing the changes in morphology, electrophysiology, metabolism, and cell cycle progression. Subsequently, we delved into various signaling pathways that regulate cardiomyocyte proliferation and survival, such as Hippo–YAP, NOTCH, Wnt, and neuregulin1 (NRG1)–ErbB. Second, we also summarized the potential of epigenetic modifications (such as chromatin accessibility, histone and DNA methylation, and RNA methylation) to regulate cardiomyocyte regeneration. Finally, we explored a variety of current therapeutic strategies, including exogenous cell transplantation, endogenous cell reprogramming, and engineered targeted delivery systems, and clarified their translational capabilities and future challenges. Overall, our comprehensive work contributes to a holistic understanding of the pathophysiological alterations in heart development and regeneration, the intricate interplay of multiple signaling regulatory mechanisms, and potential clinical intervention strategies. It also provides perspectives and insights for future research on how to accurately assess and effectively control the proliferation rate of cardiomyocytes, as well as investigations into dedifferentiation and redifferentiation stages and the drug screening platforms.

## 2 Properties Change of Cardiomyocytes During the Developmental Process

From the embryonic stage to birth and into adulthood, the proliferative capacity of cardiomyocytes significantly declines. Compared with adult cardiomyocytes, embryonic cardiomyocytes exhibit distinct differences in cellular morphology, structure, metabolic pathways, electrophysiology, and cell cycle characteristics. An in‐depth investigation and intervention of these features may lead to the identification of critical targets and methods to promote cardiomyocyte proliferation.

### 2.1 Morphological Change

In mice and other mammalian embryos, cardiac growth primarily occurs through the proliferation of cardiomyocytes, whereas after birth, it gradually shifts to a hypertrophic growth pattern to increase heart size [22]. Fetal cardiomyocytes have small volumes and are typically round or rod shaped. During development, cardiomyocytes interact with each other, forming functional syncytia through gap junctions and adhesion molecules, ultimately developing into mature adult cardiomyocytes with large, anisotropic and long rod‐shaped morphology [23, 24]. As for myofilament proteins, in fetal cardiomyocytes, sarcomeres are typically smaller, and myofibrils are arranged in a disorganized manner scattered across the cytoplasm. During development, sarcomeres gradually increase in thickness, occupying most of the cytoplasm, and the structure becomes more ordered, with clear visible Z‐lines and intercalated discs. The formation and organization of sarcomeres also depend on the expression of different myosin proteins. During development, the TTN gene (encoding titin) messenger RNA (mRNA) undergoes extensive alternative splicing; adult cardiomyocytes express the short and stiff isoform N2B of titin, while embryonic cardiac muscle cells express the longer and more elastic isoform N2BA of titin [25, 26]. Moreover, fetal cardiomyocytes of both mice and rats lack T‐tubules, accompanied by the spatial decoupling of L‐type calcium channel and ryanodine receptors leads to delayed calcium‐induced calcium release, resulting in weaker cell contractile force [27]. After birth, T‐tubule‐like subcellular structures gradually appear in the submembrane region of cardiac myocytes, and mature completely around 1 month [28]. Fetal cardiomyocytes also exhibit smaller mitochondria, lower mitochondrial content, and fewer mitochondrial cristae. These structural features result in decreased mitochondrial oxidative phosphorylation and intracellular reactive oxygen species (ROS) levels, which are beneficial for ensuring the integrity of DNA [29].

### 2.2 Electrophysiological Change

**FIGURE 1:**
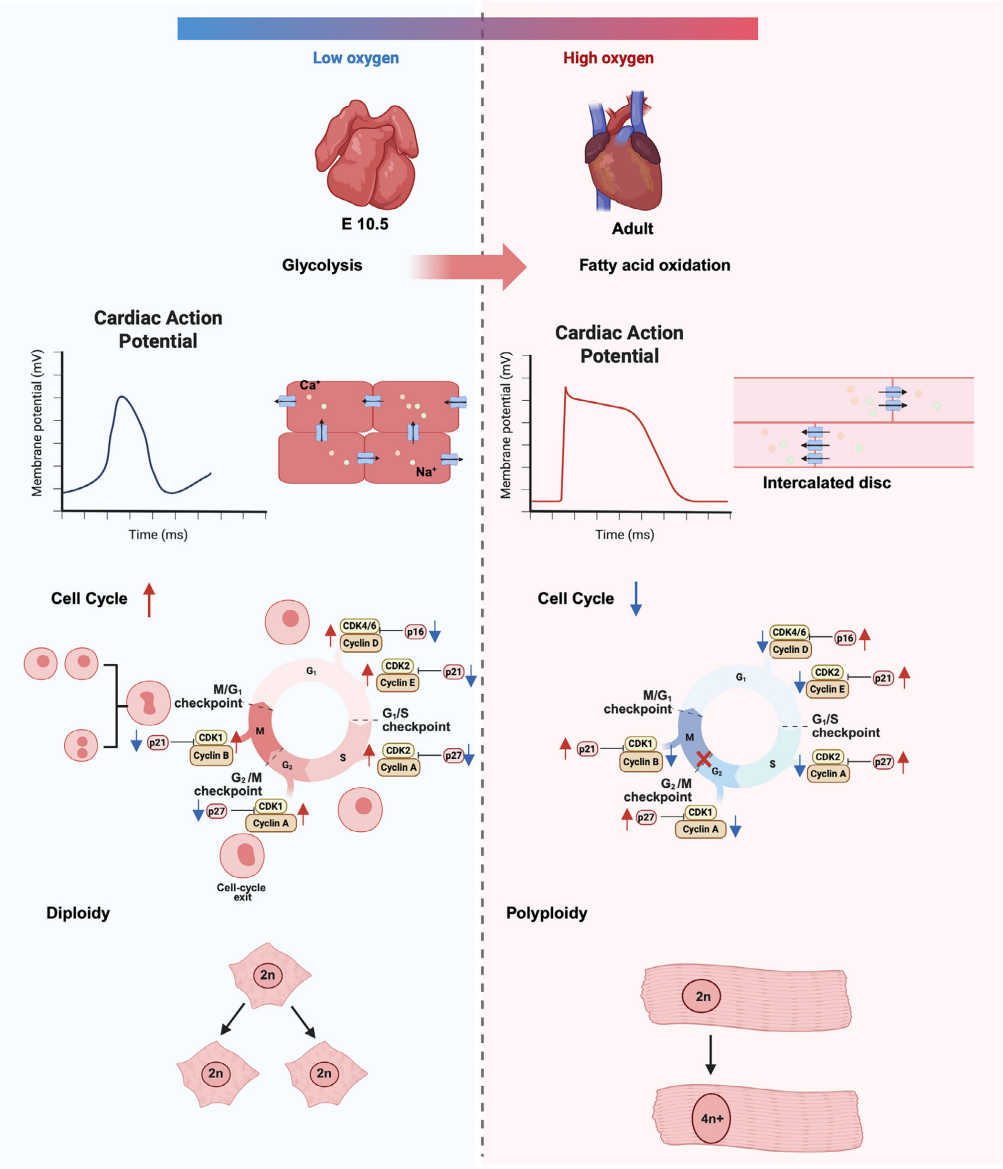
Schematic illustrating the key transformations cardiomyocytes undergo during development. As oxygen exposure increases, cardiomyocytes exhibit morphological adaptations—from a low‐oxygen fetal configuration to a mature high‐oxygen adult structure. Electrophysiologically, fetal cardiomyocytes have shorter action potentials mediated by Na⁺ and Ca^2^⁺ channels, while adult cardiomyocytes develop prolonged action potentials and intercalated discs for efficient electrical signaling. Metabolically, there is a shift from fetal glycolysis to adult fatty acid oxidation. In terms of cell cycle dynamics, fetal cardiomyocytes are proliferative (cell cycle ↑), but adult cardiomyocytes withdraw from the cell cycle (cell cycle ↓) and undergo polyploidization (2n to 4n+). Image created with BioRender.com with permission.

The excitation–contraction coupling mechanism triggered by action potentials in cardiomyocytes exhibits extremely high efficiency in adult cardiomyocytes, requiring highly coordinated activity of various ion channels and ion pumps on the cell membrane and sarcoplasmic reticulum membrane. Due to the expression of hyperpolarization‐activated cyclic nucleotide‐gated channel 4 (HCN4), immature cardiomyocytes in the early embryonic stage still possess autonomic rhythmicity. However, in the late developmental stage, HCN4 expression is confined to sinoatrial node cells to control the cardiac rhythm, and adult cardiomyocytes no longer express HCN4 [30]. The contraction of these cells is regulated by the sinoatrial node, also known as the “voltage clock,” which transmits the initial electrical signals [31]. Specifically, compared with fetal cardiomyocytes, adult cardiomyocytes have significant differences in all indexes of action potential. Adult cardiomyocytes have lower resting membrane potential, faster depolarization rate, and longer‐lasting plateau after depolarization [30, 32], as is also shown in Figure 1. The reasons for the differences may be that the ion channels involved in action potential conduction on the cell membrane and sarcoplasmic reticulum membrane are different types [33].

The transient change of calcium ion concentration that mediates action potential and myotome contraction (calcium transient) is also more rapid and efficient in adult cardiomyocytes. In other words, adult cardiomyocytes have a stronger calcium processing capacity, which is reflected in higher calcium storage in the sarcoplasmic reticulum, efficient calcium‐induced calcium release mediated by T tubule structure, and a more rapid and regulated calcium pump by sarcoplasmic/endoplasmic reticulum Ca2^+^ ATPase (SERCA2)‐mediated calcium recovery [34].

### 2.3 Metabolic Substrate Change

Multiple omics studies have confirmed that cardiomyocytes in mammals, including rodents, rabbits, and pigs, undergo “metabolic reprogramming” after birth, that is, switching energy metabolism from glycolysis to fatty acid β‐oxidation (FAO) (Figure 1). This change is consistent with the cardiomyocyte cell cycle withdrawal [35]. In the neonatal heart, oxygen is less needed, and glycolysis accounts for about 50% of total adenosine triphosphate (ATP) generation in the heart at P1, decreasing to 10% by P7 and 5% by adults. FAO provides 10% of total ATP production in the heart at P1, increasing to approximately 50% by P7 as the heart's demand for oxygen and energy increases, and increasing to >90% by adults [36, 37]. Although amino acids are not the main energy source for cardiomyocytes, they can also be utilized for energy under conditions of prolonged fasting, intense or prolonged exercise, and certain pathological states. Metabolomics studies have found that hat P1 had higher glutamine and kynuuric acid levels than P7 cardiomyocytes [38, 39]. In addition, the metabolism of histidine, serine, and threonine is more active, while the metabolism of S‐adenosylmethionine and taurine is reduced in proliferating cardiomyocytes [40]. However, it is not yet clear whether these changes are related to heart regeneration. It should be pointed out that blood oxygen, metabolic substrates, hemodynamic stress, hormone levels, and breast milk may work together to trigger metabolic reprogramming in tandem with cell cycle withdrawal. At present, the mainstream view is that metabolic reprogramming may be the initiating factor rather than the byproduct of cardiomyocyte proliferation [39, 41, 42].

### 2.4 Cell Cycle and Ploidy Change

Mononuclear diploid cardiomyocytes (MNDCMs) are generally considered to be proliferative, whereas binuclear or multinuclear polyploid cardiomyocytes have a weak proliferative capacity. The generation of multinucleation in cardiomyocytes is usually due to the decoupling of the cardiomyocyte cycle and cell division after birth [43]. Cardiomyocytes completed nuclear division but failed to finish cytokinesis. This process occurs in the final stages of cytokinesis, when the cytokinetic ring contracts and forms the so‐called midbody structure. The position of these midbodies ultimately determines whether the cell becomes a binucleated cell (asymmetric midbody) or completes cytokinesis fully (symmetric midbody) [44]. MNDCNs enter the cell cycle more frequently than polyploid cardiomyocytes and respond to cell cycle regulatory factors at a higher rate [45, 46], as is also shown in Figure 1. Zebrafish and newt species are typical representatives, with MNDCMs being the main source of endogenous proliferation. However, human cardiomyocytes are mostly mononuclear polyploid, and considering the high ploidy level of cardiomyocytes seems to be related to the loss of cardiac regenerative ability, it is currently unclear whether these cells can divide and by what mechanism. The latest exciting research indicates that quantitative analysis of nuclear ploidy in cardiomyocytes can be achieved by measuring nuclear volume and establishing mathematical segmentation models, thereby effectively assessing the proliferative capacity of cardiomyocytes [47]. In addition, several studies have demonstrated that targeting multiple cell cycle regulators can promote the proliferation of cardiomyocytes by influencing their ploidy levels. The initiation of DNA synthesis requires the transition of cells from G1‐S phase, which is mainly regulated by CDK4. Soonpaa et al. [48] found that overexpression of the regulatory subunit cyclin D1 of CDK4 significantly increased the multinucleation of mouse cardiomyocytes. There are many other cell cycle regulatory factors involved in cell division, such as GSK, cyclin D2, and c‐myc, which have led to nuclear division (mitosis). However, it is difficult to ensure the division of cells into two distinct daughter cells (cytokinesis) and subsequent survival [49, 50, 51]. These strategies often only activate less than 1% of cardiomyocytes, severely limiting their efficacy. Therefore, subsequent studies have found that the combination of CDK1/CCNB and CDK4/CCND, these four cell cycle regulators, can efficiently promote the proliferation of human iPSC‐derived cardiomyocytes, with an amplification rate of 20%, and the cells can still survive after division [45].

## 3 Molecular Mechanism

### 3.1 Pathophysiological Process in Heart Regeneration

#### 3.1.1 Energy Metabolism

Compared with adults, the fetal heart is situated in a relatively hypoxic environment, characterized by low circulating fatty acid levels. During this period, the energy supply primarily relies on anaerobic glycolysis and lactate oxidation, which is sufficient to meet the energy demands imposed by the relatively low workload of the heart [52]. Following the initiation of the first breath after delivery, there is a rapid increase in the partial pressure and saturation of oxygen in the blood, accompanied by a decrease in the levels of glucose and its derivatives within the circulation, while the levels of fatty acids rise. Concurrently, in order to meet the substantial ATP demands associated with increased workload, the heart begins to utilize fatty acids as its primary energy substrate [40, 53]. During this process of oxidative phosphorylation, a significant amount of ROS is produced, which can damage DNA and lead to cell cycle arrest in cardiomyocytes [35]. This phenomenon is also considered one of the critical factors contributing to the inability of cardiomyocytes to regenerate. Multiple omics studies have shown that changes in enzymes related to glucose and lipid metabolism are important causes of postnatal metabolic changes in mammals, such as GLUT1 and PKM2 gradually decreased after birth, while CPT1 and ACSL expression levels increased [52, 54, 55]. By interfering with the gene expression of these key metabolic enzymes, the proliferation of cardiomyocytes can be promoted in vitro and in vivo. Enhancing the expression of enzymes related to glucose metabolism is one of the key factors in activating heart regeneration capacity.

In the context of regulating glucose metabolism, GLUT1 is the main glucose transporter expressed in the embryonic and neonatal heart, and studies have shown that overexpression of GLUT1 in the heart leads to increased glucose uptake, glycogen storage, nucleotide content, and enhanced heart regeneration and repair capabilities [56]. PKM2 is an isoenzyme of the glycolytic enzyme pyruvate kinase that regulates the conversion of phosphoenolpyruvate and adenosine diphosphate to pyruvate and ATP. Specific knockout of Pkm2 during cardiac development can lead to cell cycle arrest in cardiomyocytes and a reduction in the number and size of cardiomyocytes. Furthermore, administration of cardiac‐specific PKM2 mRNA following acute or chronic MI in mice can enhance cardiomyocyte proliferation, improve cardiac function, and increase long‐term survival rates [55]. Lactate dehydrogenase A (LDHA) is an enzyme that catalyzes the conversion of pyruvate to lactate at the terminal stage of glycolysis. In adult mice, cardiomyocyte‐specific overexpression of LDHA leads to a reduction in various intermediates of the tricarboxylic acid cycle, enhances glycolysis, and additionally induces the polarization of macrophages toward the M2 phenotype, thereby alleviating the production of ROS and creating a microenvironment conducive to heart regeneration [57].

On the other hand, the inhibition of certain enzymes involved in promoting FAO and the TCA cycle can also effectively stimulate the proliferation of cardiomyocytes. CPT1 is the rate‐limiting enzyme of FAO, responsible for regulating the transport of acyl‐CoA across the inner mitochondrial membrane into the matrix. Both the inhibition of fatty acid metabolism in neonatal rats using the CPT1 inhibitor Etomoxir and the provision of fatty acid‐deficient milk to newborn mice can extend the window period during which cardiomyocytes exit the cell cycle [58, 59]. More intriguingly, the embryonic stage‐specific knockout of the CPT1b gene can effectively induce metabolic reprogramming, inhibit the utilization of long‐chain fatty acids, and promote hypertrophic growth of the heart. Furthermore, the conditional knockout of the CPT1b gene in adult cardiomyocytes can significantly enhance the proliferation of cardiomyocytes following ischemia–reperfusion (I/R) injury, resulting in the near‐complete repair scar tissue in the reperfusion area [42]. Sphingolipids, derived from serine and fatty acids, serve as crucial components on cell membranes, participating in important physiological processes within cells [60]. Li et al. [61] first revealed the significant role of sphingolipid metabolism in mammalian heart regeneration. Following myocardial injury, the levels of the core sphingolipid metabolite sphingosine 1‐phosphate (S1P) were markedly upregulated, accompanied by the upregulation of its synthesizing enzyme SPHK2. Specifically, the overexpression of SPHK2 in the myocardium markedly promoted the cardiomyocyte proliferation in postinjury neonatal mice, extended the proliferative window and simultaneously promoted cardiac regenerative repair following MI in adult mice. Ketone bodies, as alternative energy substrates to fatty acids and glucose following cardiac injury repair, primarily originate from the liver. In recent years, there has been increasing research interest in the metabolic changes of ketone bodies during cardiac development. Multiomics analysis showed that the levels of β‐HB, as well as the genes or proteins such as HMGCS2 (ketogenesis) and OXCT1 (ketone oxidation), exhibit a significant increase pattern from the embryonic to neonatal period [62].The cardiac conditional knockout of HMGCS2 in neonatal mice results in immature mitochondrial development and a significant repression of FAO, suggesting that the inhibition of ketone body metabolism may be beneficial for cardiomyocyte dedifferentiation and regeneration [62]. However, another study during the same period indicated that overexpression of HMGCS2 increased myocardial cell dedifferentiation and proliferation in adult mice and hiPSC‐CM under MI or hypoxia [63]. The above two studies present conflicting findings regarding the interplay between ketone body metabolism and fatty acid metabolism in mediating heart regeneration, suggesting that the regulation of ketone body metabolism to induce heart regeneration warrants further investigation.

Amino acid metabolism is involved in biomacromolecule synthesis and cardiac energy metabolism. Except for histidine, serine, threonine and lysine, the levels of most amino acids increase after birth (P04–P09) and then decrease to a stable level at P23 [64]. Glutamine has been shown to be a key amino acid for heart regeneration in zebrafish because it initiates mTORC1 pathway activation in cardiomyocytes [39]. Tryptophan (Trp) is an essential amino acid that produces various bioactive metabolites involved in the physiological regulation of the organism. Trp is metabolized by indoleamine 2,3‐dioxygenase 1 to generate kynurenine (Kyn). Zhang et al. [38] reported that the supplementation of Kyn in neonatal mice can promote the repair of damage following apical resection in pups. Kyn exerts its effects by activating the aryl hydrocarbon receptor, which influences the downstream YAP/ERK signaling pathway to promote the cardiomyocyte proliferation. Unlike other amino acids, branched‐chain amino acids (BCAAs) such as valine, leucine, and isoleucine are primarily metabolized in organs other than the liver, including skeletal muscle and cardiac muscle. They provide nitrogen for other amino acids and act as signaling molecules that activate the mTOR signaling pathway, thereby regulating cardiac homeostasis [65]. Recent research has focused on the significant regulatory roles of BCAAs (leucine, isoleucine, and valine) in heart failure. In fact, during cardiac development, the concentrations of valine, leucine, and isoleucine increase from P1 to P9, followed by a decline to levels at or below those observed at P1 by P23 [64]. This pattern was in line with a reduction of protein synthesis at P23 and an increase of fatty acid metabolism after P1. Therefore, BCAAs may also play a potentially important role in heart regeneration, and the field of amino acid metabolism may be one of the focal points for future research in cardiac regenerative medicine. In addition, the key drivers and mechanisms through which the aforementioned metabolic pathways influence cardiomyocytes proliferation are summarized in Table 1.

**TABLE 1:** The key metabolic factors and mechanisms of heart regeneration.

Metabolic pathway	Key effectors	Mechanism	Effects	References
Glucose metabolism	GLUT1 overexpression	Promotes nucleotide biosynthesis	Glycolysis↑	[56]
	Pkm2 modified RNA	Regulates the cardiomyocyte cell cycle and reduces oxidative stress damage through anabolic pathways and β‐catenin	Glycolysis↑	[55]
	HIF‐1α/LDHA inhibition	Shifts glycolysis to oxidative metabolism, reduces lactate accumulation, enhances mitochondrial maturation	Glycolysis↑	[57]
	5SM	Lactate signaling synergistically activates the mTOR pathway	Glycolysis↑	[189]
Lipid metabolism	Cpt1b inactivation	Promotes α‐ketoglutarate accumulation and leading to activation of the α‐ketoglutarate‐dependent lysine demethylase KDM5	FAO↓	[42]
	Sphk2 overexpression	Inhibits the deacetylase activity of HDAC, and increase the H3K9/H3K27 acetylation levels of Erbb4, Mef2a, and Mef2c	Sphingolipid metabolism↑	[61]
				
Ketone body metabolism	HMGCS2 knockout	Enhances acetylation of mitochondrial proteins, leading to the inhibition of the enzyme activity in mitochondria and cardiomyocyte immature	Ketogenesis↑	[62]
	HMGCS2 overexpression	Associates with PPARα for inducing SRC expression	Ketogenesis↑	[63]
Amino acid metabolism	Kynurenine	Activates the cytoplasmic aryl hydrocarbon receptor–SRC–YAP/ERK pathway	Tryptophan metabolism↑	[38]
	Elevated glutamine	Activates mTOR signaling	Glutamine metabolism↑	[39]

#### 3.1.2 Inflammatory and Immunological Responses

The immune system‐mediated inflammatory response plays a positive role in the myocardial injury repair. In the absence of initial inflammation, MI can lead to severe left ventricular dysfunction. Appropriate inflammation can facilitate endogenous tissue repair following injury. However, the persistence and exacerbation of the inflammatory response will result in adverse tissue remodeling and deterioration of cardiac function, thereby impeding the healing process [66]. In fact, previous research has indicated that the Ly6^+^ inflammatory leukocytes rapidly accumulate at the site of injury undergoing apical resection. The expression of inflammatory markers IL‐6, IL‐1β, and CCL3 would remain significantly elevated for a duration of 7 days. The administration of dexamethasone via intraperitoneal injection induces an immunosuppressive effect, resulting in incomplete regeneration of the mouse heart, which instead develops fibrotic scars at the site of the apical incision [67]. This suggests that the acute inflammatory response is essential for promoting heart regeneration.

In the context of the innate immune system, within 6–24 h after AMI, neutrophils first infiltrate into the infarct area, releasing matrix metalloproteinases (MMPs), elastase (NE), and other substances to remove dead myocardial cells as well as matrix fragments [68, 69]. However, in the process of myocardial repair, the current mainstream view tends to believe that the phagocytosis and clearance of neutrophils mainly exert negative effects. Because once the activity of neutrophils is prolonged, the phagocytic action will be enhanced, activated neutrophils release complement proteins, MMP9, myeloperoxidase, and NE through degranulation, generating a large amount of ROS, forming neutrophil extracellular traps (NETs), and so on, inducing myocardial necrosis and extracellular matrix (ECM) degradation, leading to irreversible myocardial damage [70]. Therefore, inhibiting the activity of neutrophils, reducing the aggregation of neutrophils in AMI, and regulating the inflammation caused by neutrophils may create a certain microenvironment for heart regeneration [71, 72]. Unfortunately, there is currently no direct evidence of the relationship between neutrophils and heart regeneration.

The latter half of the early phase of AMI is characterized by the rapid recruitment of monocytes from the bone marrow and spleen to the injured myocardium. Under the stimulation of chemokines, these monocytes differentiate into macrophages. The monocyte/macrophage population has been shown to play a significant role in heart regeneration following AMI. Specifically, after P1 MI, the use of chlorphosphonate liposomes to treat macrophages has been shown to inhibit heart regeneration, promote the formation of fibrotic scars, and significantly suppress cardiac function [73]. This lack of regeneration is attributed to indirect effects on vascular regeneration rather than a direct effect on cardiomyocytes. However, such strategies target general phagocytes and lack specificity; therefore, these results require further investigation. Moreover, different macrophage depletion strategies may yield varying effects [74]. Consequently, it is crucial to utilize genetic models to study the impact of depleting specific immune cell subsets or intervening in macrophage‐specific target genes on heart regeneration in neonatal mice. Research has shown that MHCII^low^ CCR2^−^ macrophages isolated from neonatal hearts possess reparative properties, stimulating cardiomyocyte proliferation and vascular regeneration, with minimal capacity to induce inflammatory responses. In contrast, CCR2^+^ macrophages isolated from adult hearts exhibit robust inflammatory cytokine response to LPS, unable to stimulate cardiomyocyte proliferation and vascular regeneration [75]. Therefore, these studies suggest that resident MHCII^low^ CCR2^−^ macrophages are key mediators of heart regeneration. In addition, studies have shown that specific deletion of oncostatin M (OSM) in macrophages inhibits heart regeneration in neonatal mouse hearts following myocardial injury [76]. Macrophages can secrete OSM, and OSM binds to the OSM receptor heterodimer OSMR/gp130. Subsequently, gp130 activates Yes‐associated protein (Yap) through Src, thereby initiating cardiomyocyte proliferation to promote heart regeneration.

**FIGURE 2:**
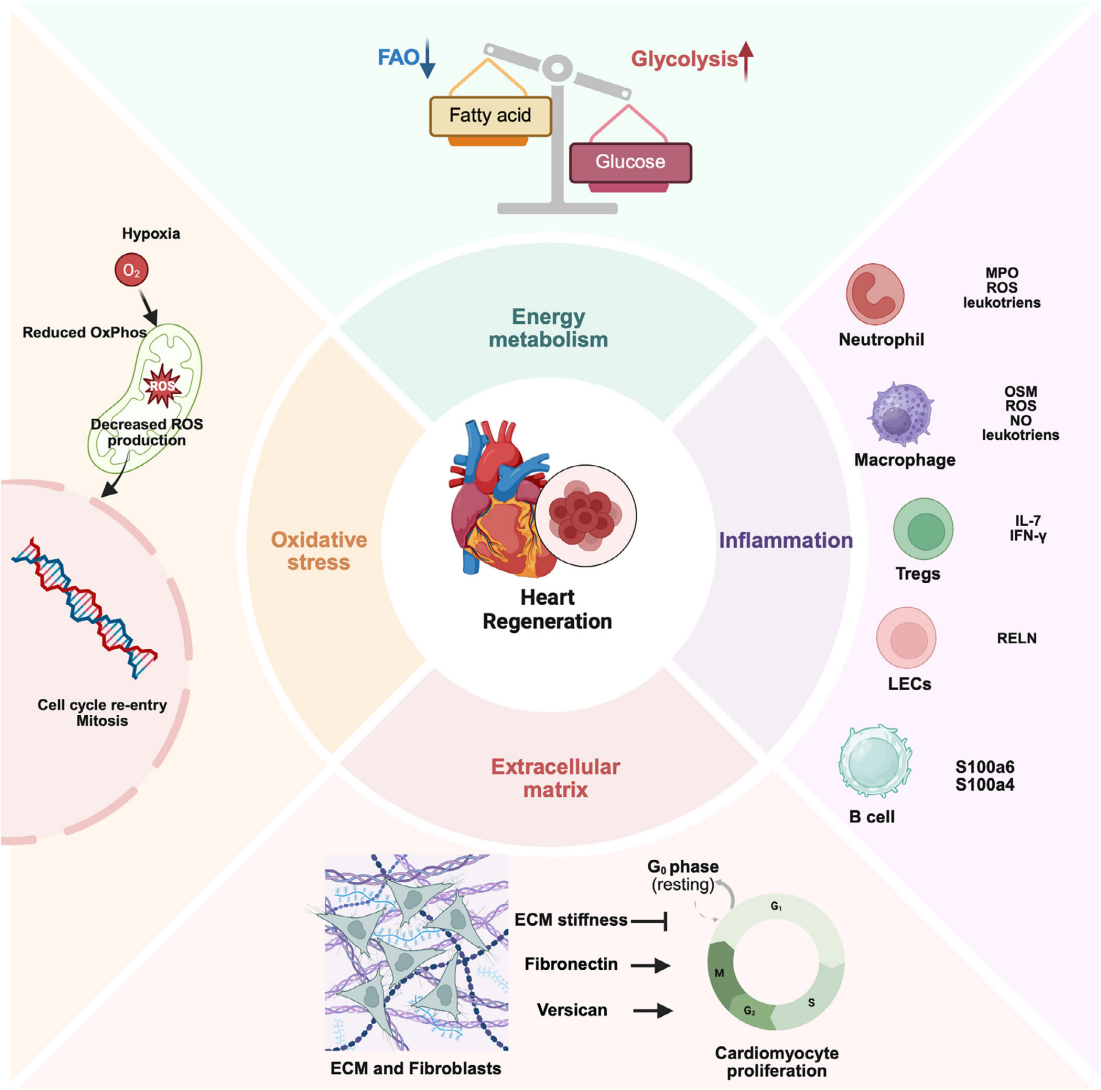
Schematic illustrating the pathophysiological process in heart regeneration. (1) Metabolic shift: During heart regeneration, cardiomyocytes preferentially utilize glycolysis over fatty acid oxidation for ATP production. This metabolic shift is particularly evident in ischemic or repair settings, where glycolysis offers a rapid energy source despite its lower efficiency, ensuring energy supply under reduced oxygen availability. (2) Immune‐mediated inflammation: The immune system plays a constructive role in myocardial injury repair. The figure highlights the involvement of various immune cells, including neutrophils, macrophages, regulatory T (Treg) cells, and B cells. Neutrophils and macrophages release inflammatory mediators such as ROS and leukotrienes to initiate repair. Macrophages further secrete cytokines like IL‐7 and IFN‐γ to promote cell proliferation and tissue restoration. (3) Oxidative stress and DNA protection: The figure indicates that proliferating cardiomyocytes under hypoxia produce less ROS, which helps reduce DNA damage. In hypoxic conditions, reduced mitochondrial respiration decreases ROS generation, while antioxidant defenses, such as heat shock proteins and antioxidant enzymes, are activated to further protect cells from oxidative damage. (4) ECM microenvironment regulation: The composition and physical properties of the extracellular matrix (ECM), including components like fibronectin and versican, significantly influence cardiomyocyte cell‐cycle progression and heart regeneration. The figure illustrates that ECM interacts with fibroblasts and secretes specific factors (fibronectin and versican) to regulate cardiomyocyte behavior, promoting heart regeneration by modulating cell cycle transitions from the resting (G0 phase) to proliferative phases (G1, S, G2, M). Image created with BioRender.com with permission.

The adaptive immune system is also a component of the proinflammatory environment in the heart following injury. It plays a multifaceted role in heart repair and regeneration, a subject that has only recently begun to receive attention and ongoing elucidation. Among the various types of lymphocytes, regulatory T (Treg) cells represent a distinct category of lymphocytes that play a crucial role in anti‐inflammatory responses and the maintenance of immune homeostasis. Due to their significant functions in promoting the repair of bone, skin, nerves, and blood vessels, the role of Tregs cells in heart regeneration has also been partially elucidated. Zacchigna et al. [77] detected proliferating cardiomyocytes in the hearts of pregnant mothers, while Treg cell depletion during pregnancy can decrease the proliferation of maternal and fetal cardiomyocytes. Following AMI, Treg cells depletion leads to decreased cardiac function, massive infiltration of inflammatory cells, and reduced collagen deposition in the scar. Injection of Treg cell can reduce infarct size, maintain contractility, and increase the number of proliferating cardiomyocytes. This may be closely related to the paracrine promotion of fetal and maternal cardiomyocyte proliferation by Treg cells [78]. Furthermore, there exists a unique lymphatic structure within the heart, which has been demonstrated to regulate fluid homeostasis and facilitate immune surveillance and clearance, thereby playing a crucial role in maintaining cardiac function and promoting myocardial healing following injury [79]. Current research on the mechanisms by which cardiac lymphatics promote cardiac repair following injury primarily focuses on two aspects: lymphangiogenesis and the secretion of lymphatic endothelial cells (LECs). Cardiac injury can induce lymphatic vessel formation response, while zebrafish mutants without lymphatic vessels exhibit severely impaired regenerative capacity [80]. Research conducted on mice further reveals that LECs secrete signaling molecules known as extracellular proteins—reelin—which play a crucial role in regulating the proliferation and survival of cardiomyocytes during cardiac development [81]. This signaling not only enhances cardiac regenerative capacity in neonatal mice but also provides protective effects for the heart following MI. Regarding other immune cells, it is noteworthy that there is relatively limited research on B cells. B cells exhibit diametrically opposed roles in heart regeneration between neonatal and adult mice. In neonatal mice, myocardial B cells are essential for cardiomyocyte proliferation and heart regeneration. Single‐cell RNA sequencing reveals that B cells in the hearts of P1 mice possess a significantly enhanced capacity to suppress inflammatory responses and promote angiogenesis compared with those in P7 and adult mice. Conversely, the absence of B cells in adult mice has been shown to inhibit tissue inflammation, reduce myocardial fibrosis, and improve cardiac function. Furthermore, the proportion of cardioprotective B cell clusters expressing high levels of S100a6 (S100 calcium‐binding protein A6) and S100a4 (S100 calcium‐binding protein A4) in the cardiac tissue of adult mice following cardiac injury is significantly reduced when compared with that in neonatal mice [82]. In summary, future targeted modulation of the immune system and downstream inflammatory responses may emerge as a viable strategy to promote cardiac regenerative therapies (Figure 2), particularly with regard to the less‐explored lymphocyte subpopulations and cardiac lymphangiogenesis and secretion.

#### 3.1.3 Oxidative Stress and DNA Damage

For decades, ROS have been regarded as harmful molecules, encompassing various free radicals derived from molecular oxygen that disrupt normal cellular pathways [83]. However, an increasing body of research indicates that redox homeostasis plays a crucial role in ensuring the self‐renewal and differentiation of cardiovascular stem cells [84, 85]. In other words, both excessive and insufficient levels of ROS can be detrimental to the cardiovascular system. Studies have shown that even low concentrations of ROS or the early upregulation of ROS are critical for promoting the maintenance of stem cells and their differentiation into cardiomyocytes. Consistently, MPTP‐mediated activation of mitochondrial oxidative metabolism plays a crucial role during the differentiation process [53]. However, as development progresses and over time, the DNA damage caused by excessively elevated intracellular levels of ROS, early studies have also confirmed that the increase in mitochondrial respiration temporally corresponds to the increase in ROS in the neonatal heart, which is also associated with an increase in oxidative DNA damage and the activation of the DNA damage response [35]. More specifically, when fatty acids relative to pyruvate are used for respiration, mitochondria produce a large amount of H_2_O_2_ at a higher rate [86, 87]. ROS can inflict damage on bases, deoxyribonucleotides, and DNA double strands, thereby triggering the DDR [35, 88]. Once activated, the DDR initiates various cell cycle checkpoints, including ATM and Chk1/Chk2, which operate at critical phases of the cell cycle [89]. These checkpoints induce a temporary halt in cell division by activating cell cycle inhibitors such as p21 and Wee1, thus providing the cell with the necessary time to repair DNA damage [89, 90, 91]. If the extent of DNA damage is too severe to be repaired, the cell may be directed toward programmed cell death or permanent cell cycle arrest [75]. Therefore, the antioxidant system plays a crucial role in maintaining cellular redox homeostasis and providing the necessary favorable microenvironment for heart regeneration during the later stages, with NRF1/2 and hypoxia‐inducible factor‐1α (HIF‐1α) serving as key effector components of this system. As a member of the Nrf family, Nrf1—an oxidative stress response transcription factor (TF) encoded by the nuclear factor erythroid 2‐like 1 (Nfe2l1) gene—was found to be significantly activated in regenerating cardiomyocytes through spatial transcriptomics analysis. Single‐cell sequencing further revealed that the deletion of the Nrf1 gene inhibits the activation of the transcriptional programs necessary for heart regeneration in neonatal cardiomyocytes. Conversely, the use of AAV9 gene therapy to overexpress Nrf1 can protect the hearts of adult mice from I/R injury. Nrf1 achieves this protective effect by activating ROS scavengers and enhancing proteasome activity, thereby maintaining the balance of oxidative and proteolytic stress [92]. Correspondingly, as a member of the same family, Nrf2 is encoded by the NFE2L2 gene. In cardiomyocytes, Nrf2 regulates the downstream target Pitx2, which responds to oxidative stress through YAP‐mediated activation of target genes, thereby protecting cells from ROS‐induced damage and promoting myocardial protection as well as postinjury regeneration processes [35, 88]. Furthermore, during embryonic development under hypoxic conditions, the TF HIF‐1α remains stably expressed in cardiomyocyte [93]. It facilitates cellular transition to a reduced state to counteract oxidative stress by mediating transcriptional alterations (such as Oct4, Nanog, and GATA4) and metabolic reprogramming, thereby sustaining cardiomyocyte proliferation [94]. In contrast, under hyperoxic conditions during the neonatal period, HIF‐1α undergoes degradation via the oxygen‐dependent ubiquitin–proteasome pathway, leading to a rapid decline in its activity postnatally [93, 95]. Maintaining a hypoxic environment after birth and stabilizing HIF‐1α can inhibit cardiomyocyte exit from the cell cycle while suppressing mitochondrial maturation, thereby promoting their proliferation [95, 96], as is shown in Figure 2. The conclusions from single‐cell sequencing indicate that the expression of genes associated with the respiratory chain and oxidative phosphorylation is diminished in a subset of proliferating cardiomyocytes [97], further corroborating the findings of the aforementioned studies.

#### 3.1.4 ECM Microenvironment

The ECM is the noncellular component of all tissues, forming a complex network of fibrillar (collagen fibers) and nonfibrillar components (composed of basement membranes, proteoglycans, and glycoproteins) within the extracellular space. In addition to providing structural and mechanical support to cells, it also transmits various biological and mechanical signals that influence different aspects of cellular behavior [98, 99]. In recent years, substantial evidence has suggested that the protein composition and mechanical properties of the ECM may play a critical role in regulating processes such as proliferation, migration, and differentiation of cardiomyocyte during heart regeneration [100, 101, 102]. During the entire process of cardiac development in mammals, the ECM transitions from a highly hydrated gel rich in morphogenetic molecules to a structurally defined collagen network that lacks such factors. Specifically, postnatally, the ECM of the heart undergoes significant remodeling, characterized by a decrease in the abundance of ECM molecules such as fibronectin (FN), hyaluronic acid, and proteoglycans, which serve as morphogenetic cues, alongside an increase in structural molecules like type I collagen, type III collagen, and laminin [103]. The ECM exhibits more organized features, which individually constrain each cardiomyocyte, thereby forming a structure reminiscent of a honeycomb. These changes correspond with the timing of the cessation of cardiac regenerative capacity. In terms of the protein components within the ECM, versican is a chondroitin sulfate proteoglycan derived from fibroblasts, primarily localized within the ECM, and is abundantly expressed during cardiac development. Research associated with versican indicates that the absence of the versican gene leads to structural abnormalities, such as ventricular septal defects, damage to the cardiac looping, and outflow tract constriction, thereby revealing its critical role in cardiac development [104]. Feng et al. [100] first discovered that versican is essential for the proliferation of neonatal mouse cardiomyocytes and heart regeneration. Moreover, the intramyocardial injection of endogenously synthesized versican can enhance the potential for cardiac repair in adult mice following MI. Notably, the structural stability of versican allows it to enter the myocardium directly without a carrier, exhibiting no immunogenicity while ensuring the safety of subsequent clinical translation. Other analogous molecules include agrin and periostin, both of which are abundantly expressed in the developing heart and have been shown to promote cardiomyocyte proliferation following MI in adult mice and pigs [105, 106]. With regard to the migration of interstitial cells, TIMP3 is a potent inhibitor that broadly targets various MMPs as well as members of the a disintegrin and metalloproteinase (ADAMs) family, all of which are involved in the degradation of the ECM [107]. Jiang et al. [101] reported that TIMP3 regulates epicardial cell migration to generate epicardium‐derived progenitor cells by remodeling the cardiac ECM, which plays a crucial role in cardiac repair and regeneration processes [108, 109]. Moreover, in the context of cardiomyocyte differentiation, the aforementioned FN is a multidomain glycoprotein composed of two subunits, each with a molecular weight of approximately 250 kDa [110]. This protein exhibits significant expression during mesoderm induction and fetal cardiac development, with its expression levels declining postnatally [111]. Research has demonstrated that FN plays a pivotal role in promoting the differentiation of human pluripotent stem cells into cardiomyocytes [112]. The aforementioned research indicates that the abundant expression of ECM proteins during developmental stages presents a promising target for reactivating heart regeneration mechanisms in adulthood (Figure 2). In addition to its composition, the mechanical properties of the ECM also influence the proliferation and regeneration of cardiomyocytes. The loss of proliferative potential in cardiomyocytes postnatally coincides precisely with an increase in ECM stiffness [113]. The injury response in conditions such as MI or other pathological states leads to extensive remodeling of the ECM, which includes alterations in its mechanical strength due to fibrotic responses. Research indicates that reducing the stiffness of the ECM can enhance the proliferation and dedifferentiation capabilities of neonatal mouse cardiomyocytes [113, 114]. Furthermore, the physical properties of the ECM can influence the efficiency of cardiac reprogramming, which is closely associated with cardiac repair [115]. Consequently, the mechanical properties of the ECM are critical for heart regeneration and repair. In both basic research and clinical applications, researchers must consider not only the biochemical characteristics of ECM components but also their physical properties. As a result, in recent years, the integration of engineering and medicine has led to the use of functionalized ECM‐based biomaterials and delivery systems, such as hydrogels or adhesive patches, to promote heart regeneration and repair, some of which have yielded promising results in clinical studies [116, 117, 118]. However, it should be noted that the aforementioned findings suggest the biological effects of ECM are likely attributable to the synergistic interplay between protein components and mechanical properties, whereas existing studies predominantly analyze these factors in isolation. For instance, whether the molecular pathways through which FN promotes cardiomyocyte differentiation are modulated by substrate stiffness remains unexplored. Such interactions have yet to be systematically elucidated, potentially compromising the precision of ECM‐targeted therapeutic strategies. Furthermore, ECM exhibits spatiotemporal dynamics during cardiac development, homeostasis, and injury repair. Following MI, for example, significant differences in composition and stiffness exist between fibrotic areas and border zones of the ECM [119], which may differentially influence regenerative outcomes. Current research predominantly employs homogenized in vitro models (e.g., two‐dimensional cultures or hydrogels with uniform stiffness), which fail to recapitulate the inherent heterogeneity of in vivo ECM and may consequently limit the generalizability of conclusions.

### 3.2 Regulation of Signal Transduction Pathways in Heart Regeneration

#### 3.2.1 Hippo and YAP Signaling Pathway

**FIGURE 3:**
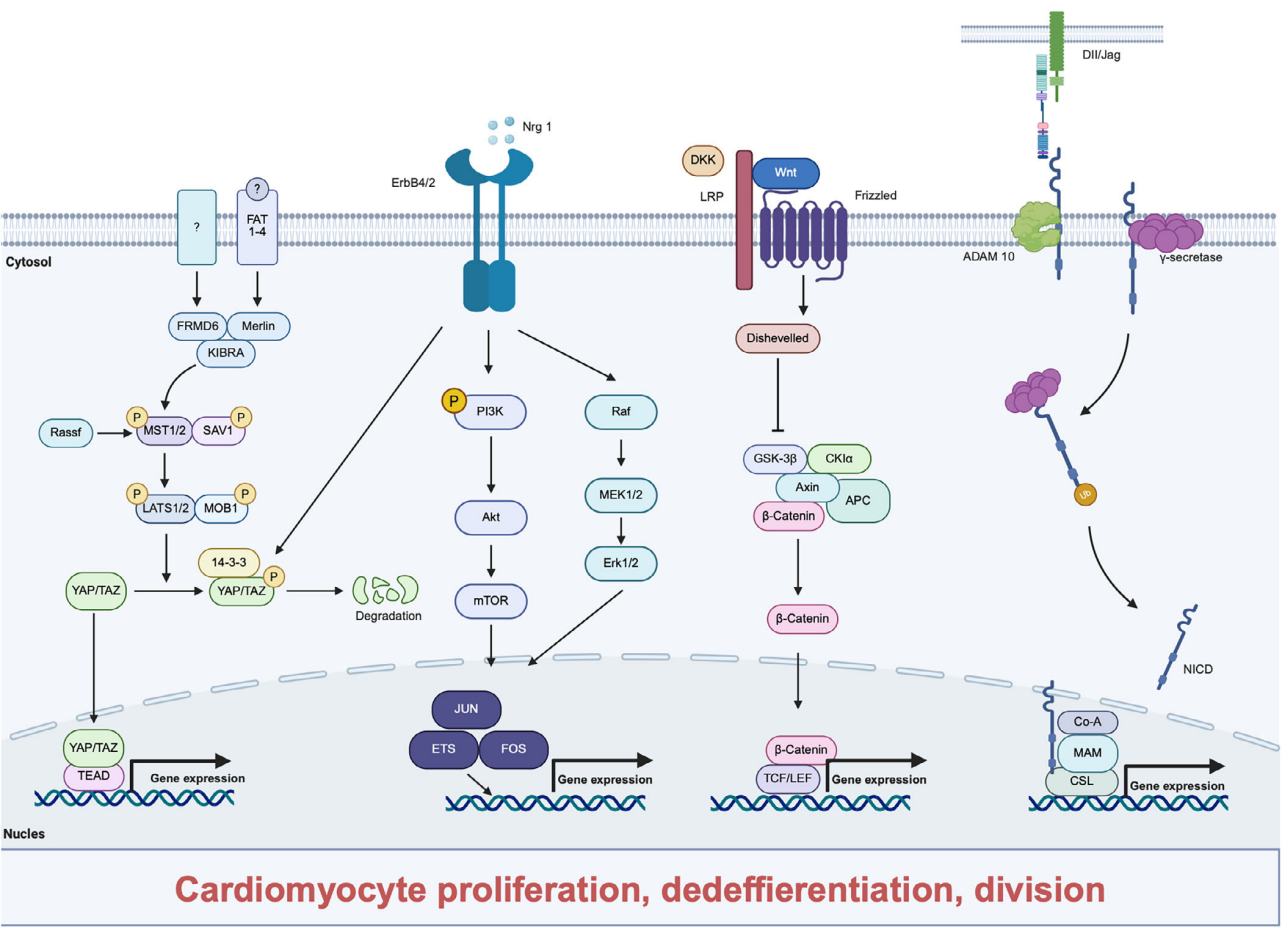
Schematic illustrating the classical signaling pathway in heart regeneration. The figure displays various signaling pathways including Hippo–YAP, Notch, Wnt, and Nrg–ErbB, which play crucial roles in regulating multiple processes of cardiomyocytes. Specifically, these signaling pathways can influence cardiomyocyte proliferation, apoptosis, division, differentiation, and maturation. The Hippo–YAP signaling pathway is shown to be involved in controlling cell proliferation and apoptosis by regulating the activity of YAP. The Notch signaling pathway is depicted as impacting cell differentiation and proliferation through interactions between Notch receptors and ligands. The Wnt signaling pathway is illustrated to be associated with cell proliferation, differentiation and apoptosis via the regulation of β‐catenin and other related molecules. Additionally, the Nrg–ErbB signaling pathway is presented to affect cell proliferation and differentiation by activating ErbB receptors. Through these complex signaling interactions, the heart regeneration process is orchestrated, with cardiomyocytes undergoing proliferation, dedifferentiation, division, and other changes to facilitate tissue repair and regeneration. Image created with BioRender.com with permission.

The Hippo and YAP signaling pathway comprises a series of protein kinases and transcriptional coactivators, which regulate cell proliferation and apoptosis, playing a critical role in embryonic development and the growth and development of tissues and organs [120, 121]. In mammals, when the Hippo signaling pathway is activated, upstream signaling inputs lead to the phosphorylation of MST1/2, which then associates with SAV. This interaction further activates LATS1/2 and MOB1A/B. The subsequent binding of these components results in the phosphorylation of downstream transcriptional cofactors YAP/TAZ, which are then sequestered in the cytoplasm through association with proteins, preventing their translocation into the nucleus and thereby inhibiting cell proliferation (Figure 3). Conversely, when the Hippo signaling pathway is disrupted or inactivated, YAP/TAZ translocate to the nucleus, where they form functional heterodimers with TEAD, initiating the expression of proproliferative and prosurvival genes, thus promoting cell proliferation [122, 123, 124]. Chromatin immunoprecipitation and RNA sequencing data indicate that YAP directly regulates the expression of genes involved in cell cycle processes, such as components of DNA replication, mitosis, cellular growth, and DNA repair mechanisms [123, 125]. Furthermore, YAP promotes cell survival by upregulating the expression of several antiapoptotic factors, including BCL‐2 and members of the inhibitor of apoptosis (IAP) family, such as survivin, cIAP1 (also known as BIRC2), and MCL1 [125]. Additionally, a genome‐wide analysis of YAP/TAZ binding targets reveals that they associate with promoters and drive the expression of genes related to stem cell pluripotency. Among these target genes are the well‐known Yamanaka pluripotency factors SOX2, NANOG, OCT4 (also referred to as POU5F1), and MYC, which can reprogram terminally differentiated somatic cells into iPSCs [126]. Therefore, YAP, as a key effector of the Hippo signaling pathway, is crucial for cardiac development and postnatal cardiomyocyte proliferation.

In mouse cardiac stem cells or fetal cardiomyocytes, early loss of YAP results in reduced cell proliferation, leading to the formation of abnormally thin myocardium, which consequently causes cardiac developmental defects and impaired contractile function [127, 128]. Conversely, overexpression of YAP in fetal cardiomyocytes can induce adult mammalian cardiomyocytes to re‐enter the cell cycle, enhancing cardiomyocyte proliferation and resulting in myocardial hypertrophy and trabecular expansion [129]. The activation of YAP stimulates the proliferation of neonatal and adult cardiomyocytes, extending the window for heart regeneration and enhancing the heart's capacity for repair following injury [130]. In summary, the primary downstream target genes regulated by Yap predominantly involve cell cycle (Aurka, Ccnb1, Ccna2, Cdc20, Cdk1) [131], cell proliferation (Myc, E2f1, and E2f2) [132], apoptosis (BCL‐2, cIAP1, MCL1), and fibrosis (IL33, IGF‐1) [133]. Furthermore, in recent years, both fundamental and translational research concerning the regulation of the Hippo signaling pathway in heart regeneration has made significant progress, while also identifying various relevant targets that influence this pathway. Specifically, macrophage‐secreted factor OSM [76], the metabolic product Kyn [38], the ECM protein Agrin [105], the growth factor receptor ErbB2 [134], the noncoding RNA miR‐199a‐3p [135], and progesterone [136] all promote cardiomyocyte proliferation through the activation of Yap. Conversely, α‐catenin [137], β1‐AR/Gαs signaling [138], and the dystrophin protein [139], which is associated with muscular dystrophy, inhibit cardiomyocyte proliferation by blocking Yap activation.

There are still several critical unresolved issues must be addressed before fully harnessing the potential of the Hippo/YAP pathway in heart regeneration. First, the potential adverse effects of transient Hippo/YAP activation in myocardial tissue and its implications for human health require thorough evaluation. Second, the specific triggers that activate Hippo/YAP in regenerative myocardial tissues, such as those observed in neonatal mice, remain unclear, as does the reason why such mechanisms fail to initiate in adult myocardial tissues. Finally, current understanding of the precise molecular mechanisms by which Hippo/YAP promotes heart regeneration remains preliminary. Thus, a deeper comprehension of the genes and biological processes regulated by this pathway may facilitate the development of novel regenerative medicine strategies that bypass direct Hippo/YAP activation (Table 2), thereby mitigating potential risks associated with its excessive activity.

**TABLE 2:** The key effector factors and mechanisms of heart regeneration.

Key effector	Mechanism	References
p38 MAPK inhibition + FGF1	Inhibiting p38 MAPK activity combined with FGF1 activates the PI3K pathway, promoting cardiomyocyte dedifferentiation and proliferation	[187]
Neuregulin 1 (NRG1)	Activates ERBB2/ERBB4 receptors, inducing mononucleated cardiomyocyte proliferation and sarcomere disassembly	[169, 170, 171]
miR‐15 family	Targets checkpoint kinase 1 (Chek1), repressing G2‐M transition and cell cycle progression	[269, 270]
lncRNA CPR	Recruit DNMT3A to the promoter region of the Mcm3 gene, facilitating DNA methylation	[210]
CircNfix	leading to the ubiquitin‐mediated degradation of Ybx1 and downregulation of its downstream target genes cyclin A2 and cyclin B1	[279]
Hippo pathway (YAP/TAZ)	YAP promotes cell cycle gene expression via TEAD transcription factors; Hippo pathway inactivation enhances proliferation	[127, 128, 129, 130]
Transcription factors (GMT/GHMT)	GATA4, MEF2C, TBX5 (GMT) ± HAND2 (GHMT) directly reprogram fibroblasts into functional cardiomyocytes	[284, 285]
Cyclins/CDKs	Cyclin A2, D2, and CDKs (e.g., CDK1/4) drive cell cycle re‐entry in adult cardiomyocytes	[45]
Thyroid hormone signaling	Suppresses cardiomyocyte proliferation via β‐adrenergic receptor‐mediated inhibition of *Ect2*; inhibition enhances cytokinesis	[261, 262]
Meis1 deletion	Downregulates cell cycle inhibitors (p15, p16, p21), enabling adult cardiomyocyte proliferation	[264, 266]

#### 3.2.2 NOTCH Signaling Pathway

The NOTCH signaling pathway is a highly conserved signaling mechanism that regulates growth and development within organisms through intercellular interactions [140]. It plays a precise regulatory role in processes such as cell differentiation, apoptosis, and proliferation, thereby exerting significant influence on the development of the nervous system, the cardiovascular system, and organ formation [141]. This pathway primarily consists of NOTCH receptors and their ligands, downstream signaling molecules, regulatory factors, and nuclear response processes. NOTCH receptors and ligands are both membrane proteins, and four homologous NOTCH receptors (NOTCH1–4) have been identified in mammals, which are widely distributed on the surfaces of various cell types, including ESCs, hematopoietic stem cells, vascular endothelial cells, and monocytes/macrophages. Additionally, there are five ligands: Jagged1, Jagged2, and Delta‐like (DLL)1, DLL3, and DLL4 [142]. The initiation of NOTCH signaling occurs through the interaction of NOTCH ligands on adjacent cells with the NOTCH receptors, followed by a three‐step proteolytic cleavage that releases a transcriptionally active fragment of the NOTCH protein, thereby activating the pathway. This cleaved fragment, which possesses a nuclear localization signal, is referred to as the NOTCH intracellular domain (ICD) (NICD). Upon its release into the nucleus, NICD binds to the TF CSL, thereby regulating the expression of downstream target genes [143], as is shown in Figure 3. In early mouse embryogenesis, the genes associated with the NOTCH signaling pathway are expressed, facilitating the development of the atrioventricular canal (AVC) and ventricles, including the external compact layer and the internal trabecular myocardium, as well as processes such as epithelial–mesenchymal transition [144].

Studies have found that abnormal expression of NOTCH1 during early embryogenesis leads to a failure in myocardial compaction, resulting in prominent trabeculations within the ventricular cavity and deep intertrabecular spaces that communicate with the left ventricular cavity. This condition is referred to as left ventricular noncompaction cardiomyopathy [145]. A study conducted in 2003 revealed that the expression of Notch receptors and ligands is upregulated in the hearts of adult zebrafish following amputation, suggesting a role for the Notch pathway in the activation of regenerative responses [146]. A decade later, further research demonstrated that Notch signaling is activated in endocardial cells, particularly in the AVC region. When Notch signaling is inhibited through the use of small molecule inhibitors, the transdifferentiation and proliferation of cardiomyocytes are compromised, resulting in failed heart regeneration [147]. Subsequent research has revealed that the activation of NOTCH in the endocardium interferes with the noncell‐autonomous activation of the Erbb2 and BMP signaling pathways in cardiomyocytes, which are responsible for cardiomyocyte reprogramming and proliferation [148]. In addition to the zebrafish heart regeneration model, an increasing body of research indicates that NOTCH signaling exhibits functional conservation during cardiac injury and repair in mammals; however, the molecular mechanisms by which it regulates heart regeneration may be more diverse and complex. The forced activation of Notch signaling mediated by AAV enhances the proliferation of newly formed cardiomyocytes in injured neonatal mice, yet fails to stimulate cell cycle re‐entry in damaged adult cardiac myocytes. The unresponsiveness of adult mouse cardiomyocytes to Notch activation and the loss of their proliferative capacity may be attributed to histone methylation modifications of Notch target genes, rendering them incapable of responding to NOTCH signaling [149]. Furthermore, in the injured adult heart, only the endocardial and epicardial cells near the infarct zone express NOTCH‐related genes, suggesting that the activation of the NOTCH pathway may occur in the endocardium [147]. The effects of NOTCH signaling activation on cardiomyocytes could be indirect, possibly mediated through effects on fibroblasts or endothelial cells, although this remains unclear. Interestingly, both NOTCH inhibition and Notch overexpression can suppress cardiomyocyte proliferation and heart regeneration, indicating that the dynamic changes in Notch activity are of greater significance [150]. Furthermore, during interpathway interactions, the inhibition of the Hippo/YAP pathway serves as a potent activator of NOTCH signaling [151], which may facilitate the maintenance of cardiomyocyte immaturity. This suggests that NOTCH functions downstream of the Hippo/YAP pathway and potentially exhibits an antagonistic effect.

In summary, the target gene repertoire regulated by the NICD–CSL complex following NOTCH activation remains incompletely characterized, particularly with respect to genes specifically expressed during regeneration that await systematic screening. Furthermore, the molecular switches governing the optimal activity window of NOTCH (where both excessive and insufficient activation inhibit regeneration) have yet to be elucidated. Finally, the clinical translation of NOTCH signaling is significantly hampered by the nonresponsiveness of adult cardiomyocytes to NOTCH activation and mechanistic conflicts in combination therapies (such as its antagonistic interaction with the Hippo pathway). Future research may require integrating single‐cell multiomics with artificial intelligence (AI)‐driven prediction to identify evolutionarily conserved Notch targets across species, coupled with the development of dynamic regulatory tools to maintain optimal Notch activity levels.

#### 3.2.3 Wnt Signaling Pathway

The Wnt signaling pathway is a highly conserved signaling cascade that plays a crucial role in various biological processes, including embryonic development, organogenesis, neurodevelopment, inflammation, and regeneration, in both mammalian and nonmammalian vertebrates. The Wnt pathway is categorized into the canonical and noncanonical pathways. The canonical pathway, also referred to as the β‐catenin pathway, operates by the nuclear translocation of β‐catenin, which forms a TF complex that initiates the transcription of target genes such as Axin2, c‐Myc, cyclin–D1, Cd44, Mmp2/9, Vegf, and others in cardiomyocytes [152, 153, 154], thereby exerting its effects. Axin2, as a component of the protein destruction complex, acts as a negative regulator of this pathway, limiting the duration and intensity of Wnt signaling [155].The noncanonical pathway primarily mediates downstream effects through RhoGTPases/JNK or Ca2^+^/PKC signaling [156].

In mouse models of MI, both the canonical and noncanonical Wnt pathways are activated [157]. However, the role of the Wnt pathway in cardiac proliferation is quite complex, as it may yield different outcomes at various developmental stages and with interventions targeting different components of this pathway. For instance, a study in zebrafish identified a novel small molecule Wnt inhibitor, termed cardionogen, which enhances the size of the embryonic heart by inducing cardiomyocyte formation. Administration of cardiongen during and after gastrulation promotes cardiomyocyte development, whereas its application prior to gastrulation inhibits cardiac formation [158]. These findings indicate that Wnt signaling exhibits biphasic and antagonistic effects in cardiac differentiation, contingent upon the specific developmental stage. Similarly, at various levels or steps of the Wnt signaling pathway, including Wnt secretion, ligand–receptor interactions, the destruction complex, or the localization within the nucleus, the intervention in Wnt signal transduction can exert bidirectional effects that either promote or inhibit cardiomyocyte proliferation. On the one hand, the study found that at embryonic day 13.5 (E13.5) in mice, cardiomyocytes with proliferative activity exhibited high expression levels of β‐catenin. The cardiomyocyte‐specific knockout of β‐catenin resulted in a decrease in the number of Ki67/PH3‐positive cardiomyocytes. This effect is associated with changes in the levels of the β‐catenin target gene cyclin D2 [159]. On the other hand, following apex resection, zebrafish can induce the expression and secretion of Wnt inhibitors such as Notumb1/Wif1 in the endocardium, as well as Dkk1/sFrp2 in the myocardium [160]. Conversely, the expression of Wnt ligands, including Wnt4a, Wnt6b, and Wnt8a, is diminished in the injured zebrafish heart. However, the expression of the atypical Wnt2bb is found to be elevated after cardiac injury [161]. The overexpression of induced Dkk1 or atypical Wnt2bb enhances cardiomyocyte generation and reduces fibrotic scarring following cardiac resection. This suggests that the inhibition of the canonical Wnt pathway is necessary to promote heart regeneration. The regulatory role of the noncanonical Wnt pathway in cardiac proliferation within mammalian models remains significantly underexplored. There is currently no clear consensus regarding whether the Wnt pathway promotes or inhibits myocardial proliferation. The prevailing view suggests that the effect of the Wnt pathway on cardiac proliferation may exhibit “temporal specificity” at different stages of cardiac development, leading to inconsistent research outcomes [156]. Moreover, several key components of the Wnt pathway possess noncanonical Wnt activity or are involved in Wnt‐independent molecular pathways. For instance, Axin and LRP6 are implicated in a multitude of Wnt‐independent cellular processes. These independent activities may influence heart regeneration processes [162, 163]. Furthermore, crosstalk between Wnt and Hippo signaling pathways exerts broad influences on cardiac development and disease‐related regeneration. Previous studies have shown that YAP, a key molecule regulating cardiomyocyte proliferation, can form a complex with β‐catenin, synergistically enhancing the activity of β‐catenin in the canonical Wnt pathway to promote myocardial proliferation during development [164]. Additionally, YAP can suppress the activation and differentiation of cardiac fibroblasts (CFs) via the Wls‐mediated noncanonical Wnt signaling pathway in cardiomyocytes, thereby modulating scar formation and heart regeneration [165].

Currently, the Wnt signaling pathway still faces multiple challenges in the field of heart regeneration. First, the cross‐interactions of the Wnt signaling pathway pose difficulties for intervention, as the network cascade effects between Wnt and pathways such as Hippo, NOTCH, and TGF‐β render single‐target strategies prone to compensatory adaptation or off‐target effects. Second, the dual functions of canonical and noncanonical pathways urgently need to be distinguished. For instance, core molecules like β‐catenin and GSK3β participate in both Wnt‐dependent and Wnt‐independent processes, yet their molecular weights in regeneration regulation remain unclear. Additionally, existing inhibitors generally lack specificity, making it difficult to avoid impacts on nontarget pathways.

#### 3.2.4 NRG1–ErbB Signaling Pathway

The neuroregulatory protein NRG1/ERBB constitutes a signaling transduction pathway formed by NRG ligands and ErbB receptors. NRG1 exerts its biological functions by inducing the dimerization of members of the tyrosine kinase receptor ErbB family, including ErbB1, 2, 3, and 4 [166, 167].The NRG1–ErbB signaling pathway plays a crucial role in maintaining cardiac developmental functions, primarily involving the participation of NRG1, ErbB2, and ErbB4 receptors [168]. NRG1, as well as the ErbB2–4 receptors, are expressed in distinct regions such as the endocardium, mesenchymal cells, and ventricular cardiomyocytes, respectively. Specific blockade of ErbB2 and ErbB4 gene expression in mouse embryos results in embryonic lethality due to underdeveloped cardiac trabeculae, indicating that the ErbB2/ErbB4 dimer is essential for the development of myocardial trabeculae; the absence of either gene leads to cardiac developmental abnormalities [169, 170, 171], as is shown in Table 2. In contrast, mice lacking the ErbB3 gene exhibit normal ventricular trabecular development but display malformations in the endocardial cushions, resulting in a high mortality rate during embryogenesis, suggesting that ErbB3 primarily influences the formation of heart valves [172]. Furthermore, mice with NRG1 knockout not only exhibit underdeveloped trabeculae but also present defects in heart valves, underscoring the critical role of NRG1 in cardiac development through the ErbB2, ErbB3, and ErbB4 receptors. NRG1 was initially reported to have mitogenic effects on cultured adult cardiomyocytes. Further confirmation in adult mice revealed that the injection of NRG1 can induce cell cycle re‐entry and cardiomyocyte division. However, the level of ErbB2 in the mouse heart declines sharply during the first postnatal week, which may render cardiomyocytes relatively insensitive to endogenous or exogenous NRG1, thereby partially compromising therapeutic efficacy.

To address this, Tzahor et al. [173] designed a transgenic system to express constitutively active ErbB2 (caErbB2) in cardiomyocytes during both the neonatal and adult periods—this mutant form represents one of the activating mutations identified in breast cancer. The expression of caErbB2 extended the postnatal proliferative and regenerative window, and persistently stimulated cardiomyocyte division from adolescence to adulthood. This mitogenic effect was so pronounced that it led to cardiac hypertrophy in caErbB2‐expressing mice, ultimately resulting in lethality. Inactivation of the tyrosine receptor ErbB4 can also diminish the proliferation of cardiomyocytes in response to NRG1 [174]. Subsequent experiments have demonstrated its most significant characteristic: the ability to induce and sustain the proliferation of adult cardiomyocytes in the absence of injury [175]. Consequently, the primary cellular target of NRG1 is likely cardiomyocytes. Research has also found that  recombinant growth factor NRG1 (rNRG1)  can induce the proliferation of cardiomyocytes in infants under 6 months of age with heart disease [176], indicating that the NRG1 signaling pathway is conserved and may serve as an effective therapeutic strategy for pediatric heart disease. However, the therapeutic potential of recombinant human NRG1 (rhNRG1) is limited in subsequent treatments due to the low expression levels of ERBB4 in adult cardiomyocytes. It is encouraging that recent research has developed a fusion protein of rhNRG1 and an ERBB3 inhibitor (rhNRG1–HER3i), which enhances the affinity between NRG1 and ERBB4, thereby promoting the proliferation of P7 and adult cardiomyocytes—an effect that rhNRG1 alone has not been able to achieve [177]. Hessel et al. [178] utilized single‐cell sequencing to further elucidate that the metabolic reprogramming of border zone cardiomyocytes, specifically toward glycolysis, is induced by the Nrg1/ErbB2 signaling pathway and is crucial for their proliferation. This novel mechanism can also be corroborated in the hearts of mice. The JK07 represents the latest generation of NRG‐1 fusion antibody therapeutics. It is the first selective ErbB4 agonist to enter the clinical development stage in the field of chronic heart failure. Currently, it is demonstrating positive outcomes in phase Ib clinical trials.

Moreover, the NRG1–ErbB can extensively interact with other signaling pathways. Research indicates that Notch signaling in the endocardium upregulates Efnb2, thereby promoting the expression of NRG1 which facilitates trabecular cardiomyocyte differentiation [179]. In endothelial cells, Yap/Taz activates ErbB2/4 receptors by regulating NRG1 expression. In mammary cells, the binding of NRG1 to ErbB receptors stimulates the cleavage of ErbB4, and the subsequently released ICD associates with Yap via WW domains (Figure 3). The Yap/ErbB4/ICD complex translocates into the nucleus [180], where it binds to TEAD TFs, thereby activating the expression of Yap‐dependent proliferation‐related genes. From this perspective, the Hippo signaling pathway may indirectly participate in regulating cardiogenesis through the NRG1/ErbB signaling cascade [181].

Similarly, the application of the NRG1/ErbB signaling pathway in heart regeneration still faces several challenges. First, the low expression of ERBB4 receptors in adult cardiomyocytes significantly limits the therapeutic efficacy of rhNRG1, although the fusion protein rhNRG1–HER3i partially overcomes this limitation by enhancing binding affinity. However, its long‐term safety and off‐target effects require further validation. Second, caErbB2 is a potent oncogenic receptor, and elucidating the critical regulatory role of its native receptor form in myocardial growth and proliferation in mice holds significant importance. Finally, at the mechanistic level, ERBB4 activation may promote fibrosis through crosstalk between the Hippo/YAP and Wnt pathways. Balancing proproliferative and antifibrotic effects remains a pressing scientific question to be addressed.

#### 3.2.5 Noncanonical and Emerging Signaling Pathways

In noncanonical pathways, the PI3K/Akt pathway regulates cellular proliferation, differentiation, apoptosis, and autophagy under both physiological and pathological conditions. This pathway is also essential for cardiac development. Activation of the PI3K/Akt pathway in MSCs leads to the overexpression of certain key components of this pathway, which have been utilized to induce the repair of infarcted cardiomyocyte and have been demonstrated to enhance the therapeutic efficacy of MSCs [182].Furthermore, the proproliferative effects of long noncoding RNAs (lncRNA) Snhg1, platelet‐derived growth factor receptor β, and the TF Tbx20 on adult cardiomyocytes are highly dependent on the PI3K/Akt signaling pathway [183, 184, 185]. The insulin‐like growth factor 1 (IGF‐1) signaling pathway activates IGF‐1R through both canonical and noncanonical routes, with IGF1R triggering two classical pathways in cardiomyocytes: the PI3K/Akt pathway and the ERK/MAPK pathway [186]. Notably, the activation of the MAPK pathway is considered an indispensable mechanism underlying the mitogenic effects of IGF‐1 [187], as is shown in Table 2. Studies have further demonstrated that IGF‐1 can inhibit necrosis in surviving myocardium, enhance cardiomyocyte function, and mitigate long‐term left ventricular dilation and remodeling [187]. Despite these findings, whether damaged mature mammalian cardiomyocytes can achieve regeneration via the IGF‐1 signaling pathway remains an ongoing area of investigation. In addition, several novel pathways have emerged that provide new perspectives for heart regeneration research. For instance, the recently identified Ctbp2–FoxO1–p21/p27 axis pathway has been shown to promote adult cardiomyocyte proliferation and cardiac repair [188]. This pathway is specifically activated under ischemic and hypoxic conditions, thereby offering a new target for the controlled and transient modulation of cardiomyocyte proliferation while avoiding the risk of myocardial hypertrophy associated with prolonged activation of traditional pathways. Furthermore, lactate signaling synergistically activates the mTOR pathway, facilitating a metabolic shift from oxidative phosphorylation to glycolysis in cardiomyocytes while enhancing cell cycle progression [189]. This mechanism can induce dedifferentiated cardiomyocytes to re‐enter the proliferative state, significantly improving cardiac function in rats following MI. YAP activation induces adult cardiomyocytes to enter a neonatal‐like state termed ACM2, while simultaneously recruiting C3AR1^+^ macrophages and C3^+^ fibroblasts via the C3 complement factor to form a “proregenerative triad.” This network remodels the postinjury microenvironment and restores the self‐renewal capacity of the myocardium [190]. In summary, these noncanonical pathways not only fill the gaps in regenerative mechanisms but also hold promises for catalyzing groundbreaking innovative therapeutic approaches.

### 3.3 Epigenetic Modification in Heart Regeneration

Epigenetic modifications refer to the chemical alterations that occur on top of DNA, extending beyond the conventional scope of genetic encoding [191]. Typically, epigenetic regulation determines the structure and accessibility of chromatin. These modifications, along with the subsequent chromatin regulation, play a crucial role in the tightly packaged DNA wrapped around histones within the cell nucleus, potentially influencing gene expression and DNA‐binding proteins [192, 193]. Consequently, they are vital in guiding cellular proliferation, fate determination, and cellular plasticity. A recent study has provided, for the first time, a comprehensive genomic view of histone coding dynamics during cardiac regeneration in zebrafish. Research indicates that after myocardial injury, there is a substantial acquisition of repressive chromatin marks on day 1, followed by the acquisition of active chromatin features on day 4, and a subsequent transition back to a repressive state by day 14. A distinct set of TFs associated with these events has been identified. The rapid transcriptional response involves the participation of super‐enhancers related to genes implicated in ECM remodeling and TOR signaling, thereby providing new regulatory targets for potentially overcoming the epigenetic barriers that impede heart regeneration. It is particularly noteworthy that there exists significant evolutionary conservation between the regulatory regions governing heart regeneration in zebrafish and neonatal mice [194].

#### 3.3.1 Chromatin Accessibility

ATAC‐seq analysis of cardiomyocytes at various postnatal time points (P1, P14, and P56) in mice reveals that the chromatin of genes associated with the cell cycle, inflammation, and ECM undergoes compaction and closure during cardiomyocyte maturation. Conversely, genes related to metabolism, muscle maturation, and contraction exhibit a transition of their chromatin from a closed to an open state [195]. These findings align with the trends in gene expression, suggesting that alterations in chromatin accessibility may underlie the changes in the expression of proliferation‐related genes. Additionally, under injury conditions, ATAC‐seq unveils variations in chromatin accessibility across different cellular subpopulations in the MI models of P1 and P8 mice: in the actively proliferating cardiomyocyte subpopulation, there is an increase in chromatin accessibility for TFs such as Tead3, MycN, and Gli1; in noncardiomyocyte populations, chromatin accessibility for factors like KLF4 in epicardial cells, FOXO4/ETS1 in endothelial cells, and EBF1/TCF21 in smooth muscle cells was significantly enhanced [196]. These results suggest the potential existence of a regulatory network between proliferative cardiomyocytes and noncardiomyocytes, wherein epigenetic regulatory mechanisms may play a crucial role (Table 3). Recent studies have further identified the cis‐regulatory elements and trans‐acting factors involved in establishing and/or maintaining noncardiomyocyte identity during the heart regeneration process in zebrafish. Specifically, it has been revealed that the downstream TF complex of TNFα signaling, activator protein 1, plays a crucial role in inducing aEpi/aFB formation and aEC activation through physical interactions with Yap1/Tead and Stat, respectively. These noncardiomyocytes are essential for heart regeneration [197]. It should be noted that while techniques such as ATAC‐seq can delineate changes in chromatin accessibility, they cannot directly demonstrate how these alterations drive gene expression or cellular behavior. For instance, the chromatin opening of proliferation‐related genes (e.g., MycN, Gli1) may merely represent an epiphenomenon of cell cycle activation rather than a deterministic factor. Furthermore, multiple cellular subpopulations (fibroblasts, endothelial cells, macrophages) exist following myocardial injury, whereas existing bulk ATAC‐seq data predominantly rely on population‐level analyses, potentially obscuring the distinct epigenetic signatures of critical subpopulations. Therefore, scATAC‐seq combined with spatial transcriptomic technologies remains necessary for further validation.

**TABLE 3:** Types, sites, and target genes of epigenetic modifications involved in heart regeneration.

Epigenetic modification	Target	Enzymes	Effect	Phenotype	References
Chromatin accessibility	Tead3, MycN, Gli1, KLF4, FOXO4/ETS1, EBF1/TCF21	None	Up	Neonatal heart regeneration	[196, 197]
Histone methylation	H3K9me3, H3K27me1/2/3H3K4me3	KAT6A, HDAC	Up or down	Cellular proliferation, cell cycle activation, formation of myofibrils	[61, 204]
Histone acetylation	H3K27ac, H3K9ac	KAT6A	Up or down	Induces immature state in cardiomyocytes and stimulates proliferation	[198, 199, 201, 202, 205, 221]
DNA methylation	CpG islands	DNMTs, KDMs	Up or down	Cardiomyocyte regeneration and differentiation	[206, 208, 228]
RNA methylation	mRNA, tRNA, and rRNA	METTL3/14, WTAP, ALKBH5, FTO, YTHDF, IGF2BP	Up or down	Cell differentiation, meiosis, and cellular proliferation and regeneration	[217, 218, 219]
Lactylation	Histone, nonhistone	P300, LDHA, KAT8	Not clear	Not clear	[57, 224]

#### 3.3.2 Histone Methylation

Depending on the specific modification sites and the genes that undergo modification, histone methylation may exert entirely opposite regulatory effects on cardiomyocyte proliferation. The tri‐methylation of histone 3 on lysine 9 (H3K9me3) has been demonstrated to inhibit cardiomyocyte proliferation. In the myocardium of adult mice, the silencing of cell cycle genes is accompanied by elevated levels of H3K9me3 modification. KDM4D (lysine demethylase 4D) is a histone demethylase targeting H3K9me3; overexpression of KDM4D in mouse myocardium can reduce the H3K9me3 levels of cell cycle genes, thereby activating the expression of these genes and promoting myocardial proliferation in adults [198]. The H3K27me3 generally leads to gene expression silencing, which has been confirmed to promote the cardiomyocyte proliferation. EZH1 (enhancer of zeste homolog 1) is a subunit of the polycomb repressive complex 2, which typically catalyzes the modifications H3K27me3, H3K27me2, and H3K27me1. Although the underlying mechanisms remain unclear, overexpression of EZH1 in mouse cardiomyocytes results in a decrease in H3K27me3 associated with cardiac development, concurrently leading to an increase in H3K27me1 levels, thereby promoting the proliferation of adult cardiomyocytes [199]. Although the study found a positive correlation between the decrease in H3K27me3 and cardiomyocyte proliferation, further research is needed to determine whether the concomitant increase in H3K27me1 directly regulates cardiomyocyte proliferation. Correspondingly, its family member EZH2 has been shown to reduce the expression of factors that inhibit cardiomyocyte proliferation, such as Lnk4a/Arf, by promoting H3K27me3 modification, which is essential for neonatal heart regeneration and cardiomyocyte proliferation [184]. However, some studies have shown that the sole inactivation of EZH2 is insufficient to affect cardiac regeneration, which may be attributed to the compensatory role of EZH1 [200]. Therefore, the dominant relationship and mutual interaction between EZH2 and EZH1 in heart regeneration require further elucidation. The presence of H3K27me3 at the muscle sarcomere/cytoskeletal protein genes promotes cardiomyocyte proliferation in zebrafish. In a zebrafish model of cardiac injury, H3K27me3 is observed at the muscle sarcomere/cytoskeletal protein genes of proliferating cardiomyocytes, leading to downregulation of these genes. Mutating histone H3K27 can inhibit H3K27me3, resulting in upregulation of muscle sarcomere/cytoskeletal protein genes, increased formation of myofibrils, and significant suppression of cell division [201]. Whether this phenomenon occurs in mammals remains unreported. The tri‐methylation of histone 3 on lysine 4 (H3K4me3) is a widely studied histone modification characterized by its association with active gene transcription. During the induced cardiomyocytes (iCMs) reprogramming process, there is a significant increase in H3K4me3 levels at the cardiac promoter, accompanied by a rapid and pronounced elevation in its mRNA expression [202]. However, recent research indicates that the knockout of CPT1b in mouse myocardium leads to elevated levels of α‐ketoglutarate (α‐KG), which in turn activates the demethylase KDM5. This activation results in a reduction of H3K4me3 levels at the promoter regions of genes associated with cardiomyocyte maturation, thereby inhibiting the expression of promaturation genes such as mylk3 and cacna1g. Consequently, this process induces a relatively immature state in cardiomyocytes and stimulates their proliferation [42]. However, it remains unclear whether other metabolites (such as succinate and NAD+) influence H3K9me3 or H3K27me3 through similar mechanisms.

#### 3.3.3 Histone Acetylation

Histone acetylation is another widely reported form of epigenetic regulation, closely associated with the enhancement of gene transcription, and has also been reported to exhibit a positive correlation with cardiomyocyte proliferation. Postnatally, the decline in cardiomyocyte proliferation capacity in mice is accompanied by the deacetylation modifications of chromatin histones [203]. Compared with P1 mice, P7 mice exhibit a significant reduction in H3K27ac levels of cell cycle and inflammation‐related genes. Intervention with lysine acetyltransferase 6A (KAT6A) to increase H3K27ac in adult mouse myocardium can facilitate the re‐entry of cardiomyocytes into the cell cycle and promote myocardial repair following MI [204]. Ji et al. [61] first demonstrate that SphK2/S1P can enhance the acetylation levels of H3K9/H3K27 and the transcriptional activity of Erbb4, Mef2a, and Mef2c by inhibiting the deacetylase activity of HDAC. This successfully stimulates adult cardiomyocytes to re‐enter the cell cycle, thereby awakening the endogenous regenerative potential of the heart. Additionally. mitochondrial uncoupling protein 2 (UCP2) has been demonstrated to sense changes in oxygen tension within the heart. Elevated levels of UCP2 can enhance acetyl‐CoA levels and histone acetylation, as well as alter chromatin‐modifying factors associated with cardiomyocyte metabolic cycles under hypoxic conditions, thereby mediating potential effects on the expression of genes involved in the cardiomyocyte cell cycle [205]. Whether there are other sites of histone acetylation modifications associated with myocardial proliferation capacity, as well as the regulatory mechanisms involved, remains to be elucidated through further research. Certainly, it is worth considering that the “crosstalk” between histone acetylation and methylation modifications may be severely overlooked in the context of heart regeneration. For instance, the balance between H3K27ac and H3K27me3 could determine the transcriptional state of genes [194], yet existing studies predominantly analyze individual modifications in isolation, lacking systematic modeling of the epigenetic modification network.

#### 3.3.4 DNA Methylation

In mammals, DNA methylation typically refers to the addition of a methyl group to the cytosine residue of CpG dinucleotides, a process catalyzed by DNA methyltransferases (DNMTs) such as DNMT3A and DNMT3B, as well as the maintenance isoform DNMT1 [206, 207, 208]. In the context of so‐called CpG islands, the methylation of cytosines within cells is often correlated with changes in the expression of adjacent genes. These CpG islands refer to regions characterized by a high density of cytosine–guanine dinucleotides, commonly found in the promoter regions of genes, which may silence gene expression by interfering with the binding of TFs or by recruiting repressive complexes [208]. The two extensively studied DNMTs, DNMT3A and DNMT3B, have been shown to regulate the expression of various genes in cardiomyocytes through methyl‐CpG binding domains, thereby altering chromatin accessibility and subsequently influencing downstream gene expression. Specifically, one study revealed that these DNMTs can modify the methylation status of critical TFs, such as MeCP2 (methyl‐CpG binding protein). MeCP2 was found to increase methylation in neonatal rat cardiomyocytes, a phenomenon not observed in adult differentiated cardiomyocytes, suggesting that MeCP2 may serve as a potential target in the mechanisms of cardiomyocyte regeneration and differentiation [209]. Further research has revealed that DNMT3A is involved in the methylation of the CpG island in the minichromosome maintenance protein 3 (MCM3) promoter, thereby inhibiting the transcription of MCM3, which is a key regulator of eukaryotic genome replication and cell cycle progression, ultimately impeding the proliferation of cardiomyocytes [210]. This study suggests that developing DNMT inhibitors may represent a novel therapeutic target for heart regeneration. However, it is crucial to consider that excessive demethylation could disrupt the functional maintenance of cardiomyocytes. For instance, DNMT3A knockout in hiPSCs impairs contractility and induces mitochondrial damage, along with deficits in lipid and glucose metabolism [211], highlighting the pivotal role of DNA methylation in preserving cardiomyocyte functional homeostasis. The core translational challenge lies in activating regenerative genes while avoiding cellular dysfunction caused by methylation imbalance. Meanwhile, the role of DNA methylation in the regulation of cardiomyocyte proliferation remains largely unknown.

#### 3.3.5 RNA Methylation

Similar to DNA, RNA molecules can also undergo methylation, particularly mRNA. A common epigenetic modification of mRNA is the methylation of adenosine at the N6 position (m6A) [212]. The m6A modification refers to the methylation occurring at the nitrogen atom in the sixth position of adenosine. In higher eukaryotes, the m6A modification is primarily recognized by the 5′‐RRACH‐3′ (R = A or G; H = A, U, or C) sequence and is highly enriched near the stop codon of mRNA and in the 3′ untranslated region [213]. The m6A modification is catalyzed by a methyltransferase complex, which includes methyltransferase‐like proteins (METTL) 3, METTL14, and Wilms tumor 1‐associated protein (WTAP). The α‐KG‐dependent dioxygenase homolog 5 (ALKBH5) and fat mass and obesity‐associated protein (FTO) act as demethylases that can reverse the m6A modification. RNA modified by m6A can be specifically recognized by m6A reader proteins from the YTH domain family (YTHDF) and IGF‐2 mRNA binding protein (IGF2BP), further regulating mRNA splicing, nuclear export, localization, translation, and stability [214]. The m6A modification plays various biological roles in mammals, including ESC differentiation, meiosis, and cellular proliferation and regeneration [215, 216]. Compared with P1 mice, the expression of m6A modification is significantly upregulated in the hearts of P7 mice. Furthermore, genes associated with proliferation‐related signaling pathways, such as Wnt and ECM receptors, are highly enriched in the context of m6A modification, suggesting that m6A plays a crucial role in cardiac development and regeneration [217]. A series of studies have demonstrated that modulating the expression of m6A methyltransferases, demethylases, or reader proteins can subsequently impact the processes of cardiac development or regeneration. The deficiency of METTL3 can impede the m6A modification of precursor miR‐143, thereby inhibiting the processing of precursor miR‐143 into miR‐143‐3p, which results in a significant increase in the expression of miR‐143‐3p target genes YAP and Ctnnd1 [218]. Correspondingly, the overexpression of Alkbh5‐mediated m6A demethylation leads to the upregulation of the reading protein YTHDF1, which can promote the translation of m6A‐modified genes, resulting in increased expression of the ALKBH5 target gene YAP [219]. Both mechanisms can facilitate the proliferation and regeneration of cardiomyocytes following myocardial injury. IGF2BP is a newly identified m6A reader protein that can inhibit the degradation of m6A‐modified mRNA. Research conducted by Wang et al. [203] indicates that IGF2BP3 is highly expressed in neonatal rat cardiomyocytes; however, it is undetectable in cardiomyocytes at the adult stage. Furthermore, the overexpression of IGF2BP3 has been shown to promote the proliferation of cardiomyocytes. In summary, the aforementioned studies indicate that m6A modification‐related proteins play a crucial role in the process of heart regeneration (Table 3). However, research in this area is still at the foundational stage. The molecular mechanisms underlying the role of m6A modification in heart regeneration, the activity and function of m6A‐related genes, their subcellular localization following specific binding to target gene mRNAs, as well as the regulation of associated signaling pathways, require further validation in future research. In this regard, a pivotal future challenge lies in elucidating the positional‐specific effects of m6A modifications on individual transcripts, particularly in understanding how m6A reader proteins translate specific m6A events into functional outcomes for heart regeneration. The establishment of both gain‐of‐function and loss‐of‐function models for these reader proteins, coupled with the development of assays to track various aspects of the life cycle of known methylated transcripts, will play an indispensable role in addressing this challenge.

#### 3.3.6 Other Types

m7G RNA methylation is a novel modification process that involves the addition of a methyl group to the N7 position of guanine (G) in RNA, mediated by the action of methyltransferases (N7‐methylguanosine, m7G) [220, 221]. Chen et al. [222] demonstrated that the mitochondrial transmembrane protein 11 (TMEM11) enhances the RNA m7G methylation activity of METTL1 through direct interaction, thereby upregulating the expression of activating TF 5 (ATF5) via the hypermethylation of ATF5 mRNA, ultimately inhibiting the cell cycle activity of cardiomyocytes. This study for the first time explores the previously unrecognized role of m7G modification in regulating cardiomyocyte proliferation, suggesting that novel therapeutic strategies based on m7G modification may be effective for cardiac repair and regeneration. In addition, in 2019, Zhao et al. [223] first demonstrate the existence of a novel modification known as lysine lactylation within monocytes, indicating that this new epigenetic modification mediated by lactate can directly regulate gene expression. The key enzyme catalyzing the production of lactic acid, LDHA, is expressed at higher levels in the hearts of P1 mice but exhibits a significant decline in adult myocardium. Overexpression of LDHA can elevate lactic acid levels, thereby stimulating the proliferation of adult cardiomyocytes [57]. This effect may be associated with the increased lactic acid levels promoting the lactylation of histone H3K18 in macrophages, inducing their polarization toward the M2 phenotype, and consequently creating a microenvironment conducive to heart regeneration [224]. Histone ubiquitination is a dynamic epigenetic modification that plays multiple roles and functions in the regulation of gene expression. Typically, histone ubiquitination occurs on histones H2A, H2B, and H3. The most extensively studied modification is the monoubiquitination at lysine 119 of H2A [225, 226]. In various cell types, the addition or removal of ubiquitin moieties is associated with genomic stability, regulation of gene expression, and DNA damage response. In murine models, it has been demonstrated that the ubiquitination of histone H2A at lysine 120, mediated by the RNF20/40 complex, regulates the maturation of cardiomyocytes and exerts a global impact on gene expression in these cells during development [227]. These novel differences in epigenetic regulation represent an untapped potential in the field of heart regeneration therapy. Although evidence remains scarce, they may ultimately provide significant resources and therapeutic approaches for patients with heart failure in the future.

## 4 Therapeutic Targets

### 4.1 Exogenous Cell Transplantation Therapy

Exogenous cell transplantation has been conducted for nearly a decade, demonstrating certain therapeutic effects in myocardial repair; however, the adverse reactions associated with its effects and the underlying mechanisms require careful evaluation.

The well‐established methods involve the in vitro induction of cardiomyocyte differentiation from ESCs or iPSCs, resulting in the generation of cardiomyocyte‐like cells. Subsequently, the iPSCs (ESC‐CMs, iPSCs‐CMs) are transplanted into the infarcted heart, where they can synchronize their contractions with the host myocardium and exhibit functional contractility [228, 229, 230]. Robust evidence from research indicates that the implantation of hESCs‐derived cardiomyocytes hESCs‐CM or iPSCs‐CMs into infarcted hearts allows for their survival within living myocardial tissue. The surviving myocardial‐like cells, known as iCMs, establish dual coupling with the original host cardiomyocytes through both membrane and electrophysiological interactions. This process contributes to a reduction in the area of MI, mitigates ventricular remodeling, improves left ventricular ejection fraction (LVEF), and prolongs ventricular ejection time [13, 228, 231, 232]. In 2001, a groundbreaking study from Professor Anversa's laboratory in the United States revealed that bone marrow‐derived c‐Kit^+^ cells could differentiate in vivo to form cardiomyocytes, thereby promoting heart regeneration. The researchers transplanted bone marrow‐derived c‐Kit^+^ cells into the hearts of mice with MI and found that 9 days posttransplantation, approximately 68% of the damaged area was covered by newly formed cardiomyocytes, indicating a significant degree of recovery from cardiac injury [10, 11]. These newly generated cardiomyocytes were derived from the transplanted c‐Kit^+^ cells, which also had the capacity to differentiate into endothelial cells and smooth muscle cells within the heart. However, subsequent studies have demonstrated that c‐Kit^+^ cells may not generate new cardiomyocytes in vivo. Notably, advanced genetic lineage tracing techniques based on Cre–loxP and Dre–rox dual homologous recombination systems, which specifically labeled c‐Kit^+^ cells in both cardiomyocytes and noncardiomyocytes, revealed that c‐Kit^+^ cells do not differentiate into cardiomyocytes to facilitate cardiac regeneration during heart injury repair [17]. Instead, their primary reparative role appears to be limited to paracrine mechanisms. These findings pose significant challenges to the original groundbreaking hypothesis regarding the pivotal role of c‐Kit^+^ cells in heart regeneration.

Cardiac myocardium tissue, in addition to cardiomyocytes, encompasses other cell types such as vascular smooth muscle cells, endothelial cells, and CFs. These cells also require regular renewal and transformation to maintain the homeostasis of the myocardium, and their source is epicardial‐derived cells (EPDCS) [233]. EPDCS are derived from epicardial cells through epithelial–mesenchymal transition and possess the potential to differentiate into arterial smooth muscle cells, arterial endothelial cells, and CFs both in vivo and in vitro [234]. In embryonic mouse hearts, EPDCs are capable of differentiating into cardiomyocytes; however, cardiomyocytes derived from EPDCs have not been observed in either normal myocardium or post‐MI myocardium [235, 236]. Cell interaction analysis based on single‐cell sequencing indicates that secreted factors originating from the epicardium regulate the regeneration of myocardial cells following injury [196]. Recent studies have demonstrated that transplantation of human ESC‐derived epicardial cells (hEPs) can improve cardiac function and reduce infarct size in mice and pigs after MI. Furthermore, it has been found that hEPs inhibit IFN‐β through paracrine mechanisms, promoting angiogenesis and lymphangiogenesis while enhancing cardiomyocyte survival [237]. However, whether hEPs activate the proliferation of cardiomyocytes in the infarcted heart remains to be further investigated.

Cardiospheres (CSs) are suspended mixed cell aggregates obtained from the digestion and culture of cardiac tissue from mammals such as humans or mice [238]. They possess clonogenic properties and the capacity for long‐term self‐renewal. CS‐derived cells (CDCs) are obtained by plating and expanding the cell aggregates derived from CSs in vitro. These cells exhibit characteristics similar to those of CSs and can differentiate into cardiomyocytes when cocultured with neonatal rat cardiomyocytes in vitro [239, 240]. Additionally, when injected into the infarcted hearts of mice, rats, or pigs, CDCs can differentiate into cardiomyocytes and endothelial cells, thereby improving cardiac function. However, due to the secretion of growth factors by CSs or CDCs that can enhance tissue function and promote tissue regeneration, such as vascular endothelial growth factor (VEGF) and hepatocyte growth factor (HGF), the paracrine effects of CDCs transplanted into the vicinity of infarcted tissue may surpass their direct role in differentiating and regenerating cardiac tissue [241]. Furthermore, the quantitative efficacy data of all representative cell transplantation therapies are also presented in Table 4.

**TABLE 4:** Quantitative efficacy data of cell transplantation therapies.

Cell type	Study model	LVEF improvement (absolute)	Infarct size reduction %LV	References
HESC‐CMs	Mouse MI	FS: 50.3 ± 2% (hESC‐CMs) vs. 43 ± 4% (controls)	NS	[231]
iPSC‐CMs	Cynomolgus monkey MI	EF: 60 ± 4.2% (iPSC‐CMs) vs. 48.82 ± 5.01 (PSC‐vehicle)	8.84 ± 1.99% (iPSC‐CMs) vs. 10.2 ± 1.46 (PSC‐vehicle)	[13]
CDCs	CADUCEUS trial	EF: 48.2 ± 10.3 (CDCs) vs. 48.2 ± 11.4 (control)	12.9 ± 6.1 (CDCs) vs. 20.3 ± 7.5 (control)	[232]
EPDCs	Rat MI	Improved 4.5 ± 3.6% in EPDCs group	NS	[233]
BMMNCs	REPAIR‐AMI trial	EF: 53.8 ± 10.2% (BMMNCs) vs. 49.9 ± 13.0% (control)	ND	[242]
MSCs + CPCs	Rat MI	FS: 30.69 ± 1.13% (MSCs) vs. 26.08 ± 1.32% (vehicle)	NS	[243]
CSCs	SCIPIO trial	EF: 42.5 ± 4.1% (CSCs) vs. 30.2 ± 2.5% (controls)	ND	[244]

Abbreviations: NS, no significance; ND, not displayed.

Although exogenous stem cell transplantation has demonstrated certain potential in heart regeneration, its therapeutic efficacy is constrained by critical issues such as cardiac‐specific immune rejection and electrophysiological dyssynchrony. Clinical studies reveal that the survival rate of unselected cardiac progenitor cells (e.g., C‐kit^+^ cells) falls below 5% at a month posttransplantation with long‐term engraftment remaining under 10% even with immunosuppressive agents like tacrolimus [245]. Additionally, the electrophysiological immaturity of transplanted cardiomyocytes introduces arrhythmogenic risks. iPSC‐CMs frequently exhibit heterogeneity in action potential duration (APD90 coefficient of variation >30%), reduced expression of calcium‐handling proteins (e.g., SERCA2a at 40% of adult cardiomyocyte levels), and disorganized polarization of connexin 43 (Cx43) gap junctions, collectively contributing to electrical conduction dyssynchrony. Large‐scale clinical data indicate that 15–20% of iPSC‐CM recipients develop nonsustained ventricular tachycardia [246]. Furthermore, challenges in cell retention and survival significantly limit therapeutic outcomes. Imaging‐based tracking demonstrates that only 0.1–2% of intracoronary injected stem cells localize to infarcted regions, with over 90% undergoing apoptosis within 72 h due to ROS burst in hypoxic microenvironments and IL‐1β‐mediated apoptotic pathways [247]. Finally, residual pluripotency in incompletely differentiated iPSCs poses tumorigenic risks, with preclinical studies reporting teratoma formation in 0.3–1.2% of transplanted cases [248].

Meanwhile, its long‐term efficacy and safety require cautious evaluation. Follow‐up data from existing clinical trials indicate a significant decline in cell survival and functional integration 6–12 months posttransplantation. For instance, hiPSC‐CMs reported an average LVEF improvement of 5.2% at 3 months posttransplantation, which attenuated to 2.1% by 12 months, with increased ventricular arrhythmia risk in some patients [245]. These findings highlight persistent challenges in long‐term cell survival and electrophysiological integration. Additionally, teratoma formation and immune rejection may further compromise long‐term outcomes. While animal studies of hiPSC‐CMs show no teratoma formation, long‐term safety assessments in human‐relevant models and improved methods to generate purified mature CMs are needed [228, 239, 249]. Karyotype instability from prolonged cell culture also raises neoplastic concerns [250, 251]. Immune rejection remains a hurdle, as most preclinical studies use immunocompromised models, masking potential immune responses. Immunosuppression improves graft survival but carries infection and cancer risks. To address this, creating HLA‐matched hiPSC banks could widen patient access to compatible cell therapies [228, 231]. In summary, these mechanistic barriers collectively hinder meaningful heart regeneration through current stem cell‐based approaches.

As for future directions in exogenous cell transplantation therapy, emerging strategies in exogenous cell therapy for ischemic heart disease focus on enhancing therapeutic efficacy through genetic engineering and cytokine modulation. CRISPR/Cas9‐mediated editing of TFs (e.g., TBX5, IRX4) enables precise differentiation of PSCs into ventricular cardiomyocytes, reducing arrhythmia risks posttransplantation [252, 253, 254]. For instance, targeted knockout of IRX4 in human PSCs improved ventricular‐specific gene expression and contractile function [254]. Modulating ion channels (e.g., KCNJ2, SCN5A) via CRISPR–Cas12a enhances electrophysiological synchronization between transplanted and host cardiomyocytes, addressing arrhythmogenic risks [255]. To enhance survival and engraftment, overexpression of antiapoptotic genes (Akt, Bcl‐2) and chemokine receptors (CXCR4) in MSCs significantly boosts ischemic tolerance and homing efficiency. For example, lentiviral‐mediated CXCR4 overexpression increased MSC retention in infarcted myocardium by threefold [256]. Immune‐compatible approaches, such as HLA haplotype‐matching iPSC banks and PD‐L1 engineering, mitigate allo‐ and xenograft rejection. Recent studies demonstrated that CRISPR‐edited PD‐L1‐overexpressing MSCs evaded immune clearance in allogeneic rat models [257]. It should also be mentioned that this field needs to consider how to promote the proliferation of adult cardiomyocytes as a means to facilitate heart regeneration, which appears to be a significant research direction.

### 4.2 Direct Induction of Endogenous Adult Cardiomyocyte Proliferation Therapy

The safest and least immunogenic option for cardiac regeneration is the utilization of existing cardiomyocytes [258]. Adult zebrafish and neonatal mice primarily maintain their capacity for heart regeneration through the proliferation of cardiomyocytes. In both adults and mice, the heart undergoes annual renewal of cardiomyocytes, albeit at a relatively low rate [14, 15, 259]. It has been demonstrated that the renewal of cardiomyocytes in adult mice originates from the proliferation of cardiomyocytes, indicating that mature cardiomyocytes possess a certain degree of proliferative potential.

As previously mentioned, the rapid decline in the proliferative capacity of cardiomyocytes following the birth of a newborn is attributed to the progressive differentiation of cardiomyocytes into terminally differentiated cells, which makes it challenging for them to re‐enter the cell cycle. Inducing cardiomyocytes to re‐enter the cell cycle represents a viable approach for promoting heart regeneration. Partial reprogramming is an innovative and promising approach within the field that can alter cellular fate by temporarily modifying epigenetic marks such as DNA methylation and acetylation, without altering the genetic sequence. In 2021, a landmark study demonstrated that local reprogramming, achieved through the combined direct action of four stem cell factors—Oct4, Sox2, KLF4, and c‐Myc (OSKM)—on endogenous cardiomyocytes in adult mice, can induce their dedifferentiation. This process stimulates the cells to enter the cell division cycle, thereby facilitating cardiac repair following MI [260]. This finding further substantiates the significant regenerative potential of adult cardiomyocytes. This approach exhibits high targeting specificity and in situ induction efficiency for proliferation. However, it is noteworthy that prolonged overexpression of “OSKM” leads to cardiac tumorigenesis and a decline in ejection fraction. Moreover, there is currently no research evidence supporting the role of “OSKM” in heart regeneration of large animals, indicating a considerable gap before clinical translation can be achieved. Besides, when exposure to hypoxic environments, the clearance of ROS, or the amelioration of DNA oxidative damage can extend the proliferative window of neonatal cardiomyocytes, potentially explaining the cycle arrest and diminished proliferative capacity observed in mature cardiomyocytes in the postnatal hyperoxic environment [261]. Based on this premise, Nakada et al. [95] gradually exposed adult mice to a hypoxic environment with an oxygen concentration of 7% to induce chronic severe hypoxemia. This intervention resulted in a reduction of ROS generation and DNA oxidative damage in cardiomyocytes and it was discovered that this hypoxic regimen could induce proliferation of adult cardiomyocytes, significantly improving cardiac function in mice with MI at 2 months of age. As a nongenetic editing approach, this method does not introduce exogenous regulatory factors and employs relatively mild physical environmental modulation. However, the prolonged duration and lower oxygen concentration involved may induce liver, kidney, and brain injuries, which are critical considerations when applying this protocol for heart regeneration induction [262]. Moreover, these studies have only been validated in mouse models, with no large animal studies conducted yet. Further exploration is needed to assess the feasibility of intermittent hypoxia or pharmacological simulation of hypoxia pathways, such as HIF‐1α agonists. A more moderate approach to improving oxidative stress and promoting cardiomyocyte proliferation is through the reduction of thyroid hormone levels in the body. Thyroid hormones are the primary regulators of energy metabolism and thermoregulation in mammals, as well as key factors in the cell cycle regulation of adult mammalian cardiomyocytes. Hirose et al. [263] induced downregulation of genes associated with the tricarboxylic acid cycle, oxidative phosphorylation, and myocardial contraction, alongside upregulation of genes related to the cell cycle and mitosis, by administering thyroid hormone receptor‐specific inhibitors, blocking thyroid hormone synthesis, and mutating thyroid hormone receptors. This intervention resulted in a significant enhancement of cardiomyocyte proliferation in mice. The findings of this study underscore the crucial role of thyroid hormones and their signaling pathways as regulators of energy metabolism in the exit of mouse cardiomyocytes from the cell cycle (Table 2). In the ovine embryonic development model, the inhibitory effects of triiodothyronine (T3) and thyroid hormone receptor (TR) on cardiomyocyte proliferation were confirmed, further validating the research findings obtained from mouse models [264]. This class of methods, by targeting the core pathways of energy metabolism, can systematically regulate the cardiomyocyte cycle, and existing thyroid hormone receptor inhibitors (such as Mx‐1) can be directly applied to drug screening. However, thyroid function suppression or deficiency may lead to systemic side effects such as metabolic disorders and myxedema, necessitating dynamic monitoring of hormone levels to avoid excessive inhibition that could affect nervous system development. The specific combination of CDK1, CDK4, cyclin B1, and cyclin D1 effectively induced cell division in postmitotic mouse, rat, and human cardiomyocytes, resulting in a significant improvement in cardiac function following acute or subacute MI [45]. The aforementioned cell cycle‐related proteins undergo rapid degradation via the ubiquitin–proteasome pathway, with protein expression lasting merely 4–5 days, thereby preventing sustained proliferation and tumorigenesis. However, the use of adenovirus in this study may lead to off‐target effects, inducing the proliferation of noncardiomyocytes (such as fibroblasts and immune cells), thereby potentially exacerbating arrhythmias or fibrosis.

Regulating key signaling proteins alongside TFs is also a viable approach. Key proteins that regulate the signaling pathways previously described, such as LRP6, NRG1, and YAP, can induce and promote the proliferation of cardiomyocytes and the repair of infarcted tissue scarring [162, 174, 231]. Notably, NRG1 has entered clinical trials, indicating its potential to increase LVEF in patients with chronic heart failure while reducing end‐diastolic volume (EDV) and end‐systolic volume (ESV) [265]. Therefore, NRG1 currently appears to have promising clinical translation prospects with the fastest advancement, yet the potential oncogenic risk of caErbB2 warrants simultaneous attention. The TF Meis1, a member of the three‐amino‐acid loop extension family of homeodomain TFs, also participates in the regulation of cardiomyocyte regeneration. Meis1 negatively regulates the cell cycle of cardiomyocytes, and its knockout can activate mitosis in neonatal mouse cardiomyocytes, thereby extending the window for cellular proliferation (Table 2). Fibroblast growth factor 10 (FGF10) may be involved in the regulation of heart regeneration by modulating Meis1 [266]. Additionally, Hoxb13, a member of the homeobox domain TF family, can act as a cofactor for Meis1, collaboratively regulating the entry of cardiomyocytes into the cell cycle [267]. In mature cardiomyocytes, Hoxb13 undergoes dephosphorylation at the Ser204 site under the regulation of calcineurin, facilitating the localization of Meis1 to the nucleus and jointly inducing cell cycle arrest. Notably, cardiomyocyte‐specific double knockout of Meis1 and Hoxb13 results in disassembly of cardiomyocyte sarcomeres, an increase in total cardiomyocyte numbers as well as mononuclear cardiomyocytes, a relative decrease in binucleated and multinucleated cardiomyocytes, and the occurrence of cardiomyocyte division and cellular proliferation [268]. Meis1 and Hoxb13 may represent novel targets for inducing cardiomyocyte entry into the cell cycle. The combination of Meis1/Hoxb13 and FGF10 may further enhance the regenerative microenvironment, and Meis1/Hoxb13 is highly expressed in cardiomyocytes, which reduces the risk of off‐target. However, it is still a frontier exploration, which is limited to the basic research stage and needs more experimental data from large animals. Furthermore, the integration of single‐cell transcriptome sequencing and chromatin accessibility sequencing has revealed that the TFs NFYa and NFE2L1 can promote cardiomyocyte division. The use of the AAV9 vector to overexpress NFYa and NFE2L1 in the myocardium significantly enhances the proliferation levels of cardiomyocytes in adult mice [97]. Despite advances in targeting key proteins to enhance cardiomyocyte proliferation, the incomplete restoration of cardiac function underscores the need for complementary strategies. Here, noncoding RNAs emerge as pivotal players, dynamically coordinating transcriptional, posttranscriptional, and epigenetic events to optimize regenerative outcomes.

Noncoding RNAs, such as microRNAs (miRNAs) and lncRNAs, are functional molecules that are largely devoid of protein‐coding capacity. In recent years, evidence has emerged suggesting that certain types of noncoding RNAs may play significant roles in regulating cellular proliferation, development, and even tissue regeneration [269, 270]. Multiple miRNAs participate in the regulation of cardiomyocyte proliferation by modulating mRNA expression. For instance, the miR‐15 family is upregulated following neonatal birth, and the supplementation of miR‐15 results in impaired cardiomyocyte proliferation [271, 272]. Conversely, the miR‐17‐92 cluster promotes cardiomyocyte proliferation [273]; intracardiac injection of cluster members miR‐19a/19b can induce cardiomyocyte proliferation, while systemic supplementation of miR‐19a/19b significantly ameliorates cardiac injury and heart failure in mice subjected to MI [274]. LncRNAs regulate cardiomyocyte proliferation through various mechanisms. From birth to adulthood, lncRNACAREL [275], lncRNACPR [210], lncRNACRRL [276], lncRNAAZIN2‐sv [277], and lncRNANPPA‐AS1 [278] exhibit increased expression in cardiomyocytes, leading to the inhibition of cardiomyocyte proliferation. Conversely, lncRNASnhg1 [183], lncRNAECRAR [279], lncRNASirt1antisense [280], demonstrate reduced expression in cardiomyocytes, promoting cardiomyocyte proliferation and regeneration. LncRNAs exert these effects partly through the lncRNA/miRNA/mRNA axis. For instance, AZIN2‐sv can sequester miR‐214, thereby preventing its downregulation of PTEN and restoring the inhibitory effect of PTEN on the PI3K/Akt pathway and cardiomyocyte proliferation [277]. Additionally, lncRNAs mediate chromatin remodeling and DNA repair through interactions with mRNA and proteins, thereby regulating cardiomyocyte proliferation. For example, lncRNACPR can recruit DNMT3A to the promoter region of the Mcm3 gene, facilitating DNA methylation modifications that suppress Mcm3 expression and the resulting DNA replication, ultimately inhibiting cardiomyocyte proliferation [210]. CircRNA is a type of noncoding RNA characterized by a covalently closed circular structure. Compared with neonatal rats, the overall expression level of circRNA in adult rat cardiomyocytes is significantly reduced. Among the circRNAs detected in the hearts of rats, mice, and humans, 10% exhibit conservation across these three species [281]. Among the highly conserved circRNAs, circNfix has been shown to inhibit cardiomyocyte proliferation. CircNfix is driven by the TF Meis1 and is predominantly expressed in cardiomyocytes, with its expression increasing after the birth of mice. CircNfix facilitates the interaction between the E3 ubiquitin ligase Nedd4l and Ybx1 (Y‐box binding protein 1), leading to the ubiquitin‐mediated degradation of Ybx1 and downregulation of its downstream target genes cyclin A2 and cyclin B1 (Table 2). Furthermore, circNfix also suppresses cardiomyocyte proliferation by sequestering miR‐214, thereby limiting the activation of β‐catenin. Knockout of cardiomyocyte circNfix promotes proliferation and repair in adult cardiomyocytes [281]. CircSamd4 is a circRNA specifically localized to mitochondria, and its expression declines postnatally. Similar to its host gene SMAD4, circSamd4 plays a positive role in the regulation of cardiomyocyte proliferation [282]. CircSamd4 recruits the molecular chaperone Vcp (valosin‐containing protein) to the mitochondria, inhibiting the opening of the mitochondrial permeability transition pore (mPTP), thereby reducing ROS and promoting heart regeneration in adult mice [283]. Noncoding RNAs have a vast array of downstream target genes and even target proteins. Consequently, this approach may confer robust multitarget regulatory capacity for cardiomyocyte proliferation, yet it also increases the likelihood of off‐target effects. Additionally, due to the short half‐life, there is currently no optimal in vivo delivery strategy. Systemic delivery often results in accumulation in the liver and kidneys, making cardiac targeting particularly challenging. Given that only a few of miRNA have entered clinical trials for heart failure treatment [284], let alone lncRNA and circRNA there remains a considerable journey ahead.

In summary, this section primarily delineates heart regeneration achieved through the modulation of endogenous cardiomyocyte proliferation, with key strategies encompassing molecular reprogramming, metabolic regulation, intervention in critical signaling pathways, and the synergistic role of noncoding RNAs. However, challenges such as incomplete functional recovery and limited proliferative efficiency underscore the necessity for complementary approaches. Emerging strategies—including in situ reprogramming of CFs or leveraging paracrine signals from immune cells—may synergize with endogenous proliferation to achieve robust heart regeneration.

### 4.3 Reprogramming into Cardiomyocytes or Indirect Regulation of Cardiomyocyte Proliferation Through Nonmyocyte Cells

**FIGURE 4:**
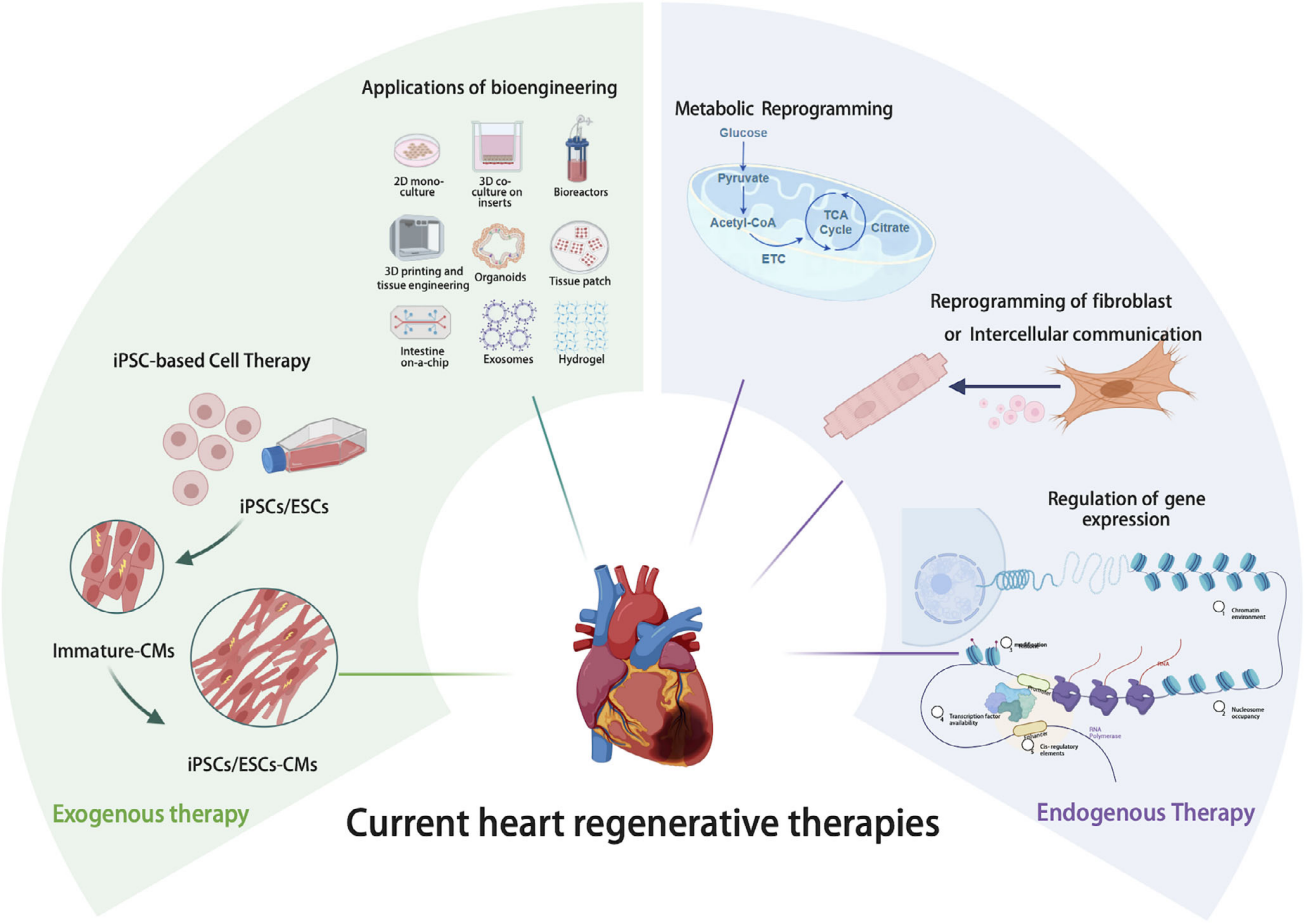
Therapeutic strategies for heart regeneration. Exogenous cell transplantation therapy involves introducing embryonic stem cell‐derived cardiomyocytes (ESC‐CMs), induced pluripotent stem cell‐derived cardiomyocytes (iPSCs‐CMs), and epicardial‐derived cells (EPDCs) into the injured heart, aiming to promote cardiac repair. In terms of biomaterials application, 3D‐printed hydrogels, engineered exosomes, are being explored for their potential benefits. For endogenous therapy, approaches such as enabling cell‐cycle re‐entry, inhibiting oxidative phosphorylation, inducing epigenetic modification, and facilitating the reprogramming and transdifferentiation of other cell types are considered viable options to stimulate cardiac regeneration after injury. Collectively, these therapeutic strategies are designed to unleash the heart's regenerative capacity, enhance cardiac function, and pave the way for innovative treatments for cardiac diseases. Image created with BioRender.com with permission.

The adult heart is primarily composed of cardiomyocytes, vascular cells, and CFs. CFs account for approximately 90% to 95% of nonmyocyte cells [285]. Therefore, if CFs could be directly programmed into functional cardiomyocytes, a substantial pool of endogenous CFs could serve as a source for regenerative therapy aimed at cardiomyocytes. Ieda et al. [286] were the first to report that terminally differentiated cells are capable of generating iCMs. Initially, they introduced 14 TFs that play critical roles in cardiac development. Subsequently, they systematically removed individual TFs from this set of 14. The results indicated that three specific TFs—Gata4, Mef2c, and Tbx5 (GMT)—were sufficient to directly convert postnatal CFs or dermal fibroblasts into iCMs in vitro. This method circumvents the iPSC stage by directly reprogramming fibroblasts into contractile cardiomyocytes that express typical cardiomyocyte markers (Figure 4). Subsequent expression of GMT in vivo via retroviral infection in mice indicates that the reprogrammed cells can differentiate into cardiomyocytes and transmit electrical signals following MI [287]. Subsequent methods have emerged to induce the conversion of fibroblasts into cardiomyocyte‐like cells. For instance, the combination of four miRNAs (miRs 1, 133, 208, and 499) with a JAK inhibitor I has been demonstrated as an alternative approach [288]. Furthermore, Lalit et al. [289] revealed that the expression of mesoderm posterior bHLH TF 1 (MESP1), GATA4, TBX5, NK2 homeobox 5 (NKX2‐5), and BAF60C in fibroblasts generates a progenitor cell population capable of differentiating into cardiomyocytes, endothelial cells, and mural cells in a mouse model of MI. Recently, Wang et al. [290] discovered that the autophagy factor Beclin1 negatively regulates fibroblast reprogramming in an autophagy‐independent manner, and that haploinsufficiency of Beclin1 in mice promotes reprogramming and reduces scar size following MI. With the establishment of various TFs, the focus in this field has now shifted toward enhancing reprogramming efficiency to improve the yield or maturity of the resulting cardiomyocytes. For instance, the incorporation of the key cardiac development gene Hand2 into the combination of three TFs known as GMT, resulting in GHMT, has been shown to significantly enhance the direct reprogramming efficiency of cardiomyocytes [287]. Furthermore, the optimization of direct cardiac reprogramming through vector delivery has been explored. Nanotechnology, with its unique targeting capabilities, can maintain the biological activity of the loaded substances, presenting promising applications in cardiac regenerative therapy. Research has demonstrated that cationic gold nanoparticles (AuNPs) loaded with GMT can serve as effective carriers for cardiac reprogramming. The AuNP/GMT/PEI nanocomplex exhibits high reprogramming efficiency and low cytotoxicity in human and mouse somatic cells, enabling direct conversion into iCMs without the need for genomic integration of the factors [291].

In addition to direct reprogramming, noncardiomyocytes can also indirectly regulate and induce cardiomyocyte proliferation through intercellular communication. Single‐cell transcriptomics and characterization of proregenerative cell states in the adult zebrafish heart have identified three transient cell states exhibiting fibroblast characteristics (col11a1a, col12a1a, and nppc fibroblasts). Notably, col12a1a fibroblasts serve as a primary source of known proregenerative genes and also express additional secreted factors with potential proregenerative functions [292]. Recent exciting research indicates that CFs contribute to myocardial repair and regeneration by synthesizing and secreting the ECM component versican. This study elucidates how versican promotes cardiomyocyte proliferation and myocardial repair by activating integrin β1 on cardiomyocytes and its downstream signaling molecules ERK1/2 and Akt [100]. Furthermore, concerning macrophages, earlier evidence suggests that heart regeneration and neovascularization following MI are dependent on neonatal macrophages. The immune phenotype and gene expression profiles of regenerative and nonregenerative cardiac macrophages indicate that regenerative macrophages possess a distinct polarization phenotype and secrete a large amount of soluble factors, which may facilitate the formation of new myocardium [73]. Subsequent research further corroborates that both the Mydgf secreted by bone marrow‐derived macrophages and the OSM secreted by cardiac macrophages can enhance the proliferative capacity of cardiomyocytes, thereby improving heart regeneration in neonatal and adult mice following cardiac injury [76, 293]. Certainly, macrophages also present potential immune‐related side effects in their future therapeutic applications. Certainly, macrophages also present potential immunological side effects in future therapeutic applications. First, the polarization state of macrophages is difficult to maintain in dynamic equilibrium. Excessive or sustained M1 activation can lead to cardiomyocyte apoptosis and fibrosis, while overactivation of M2 macrophages results in the secretion of IL‐10 and TGF‐β, which suppress T cell and NK cell activity, thereby increasing the risk of infection [294, 295]. Second, the phagocytic function of macrophages themselves may significantly reduce stem cell survival rates, thereby impairing regenerative efficacy. For instance, a recent study revealed that coculture of iPSCs with M1 macrophages reduced proliferation, cardiac differentiation, and maturation of iPSCs [296]. Furthermore, the interaction between macrophages and progenitor cells may interfere with the regenerative process. Extracellular vesicles (EVs) derived from CPCs can induce a shift in macrophages toward a proinflammatory phenotype by activating the TLR4/NF‐κB pathway, thereby suppressing the cardiac differentiation capacity of CPCs [297].

### 4.4 Engineered Targeted Delivery Induces Cardiomyocyte Proliferation

Through the interdisciplinary integration with the field of biomedical engineering, novel methods for the effective delivery of factors influencing heart regeneration have continuously emerged, including EVs, cardiac patches, and bioactive hydrogels, and photobiomodulation (PBM) among others [298, 299], as is also shown in Table 5.

**TABLE 5:** Quantitative efficacy data and comparison of engineered targeted delivery.

Therapy	Core advantages	Limitations	Key data/references
Extracellular vesicles (EVs)	Low immunogenicity; natural targeting; multitarget regulation	Unclear mechanisms; inconsistent isolation protocols; high dosage needs	hiPSC‐CM EVs improved cardiac function by 20–30% in mice [299] CD47‐modified EVs retained in myocardium for 8 h [303]
Engineered cardiac patches	Direct structural support; hybrid scaffolds (natural + synthetic materials)	Require thoracotomy; poor vascularization; low cell engraftment efficiency	Polycaprolactone/gelatin patches reduced ventricular dilation [309] Scaffold‐free MSC patches improved deep myocardial repair [314]
Hydrogels	Injectable minimally invasive; high biocompatibility; clinical progress (alginate in FIM trial)	Low mechanical modulus; poor conductivity; unknown immunogenicity	Conductive hydrogels restored electrical conduction by 40% [324] Alginate hydrogel improved LVEDV/LVESV by 15–20% [330]
Photobiomodulation (PBM)	Noninvasive; adjustable wavelength; synergizes with other therapies	Limited light penetration; unclear mechanisms; preclinical stage	Red light reduced infarct size by 30% in rats [335]

#### 4.4.1 EVs for Heart Regeneration

Lipid‐bound EVs originate from the plasma membrane or endosomal membrane and are classified into microvesicles and exosomes based on their physical characteristics, molecular markers, and functions. They possess the potential for drug delivery and contain nucleic acids (mRNA, miRNA) and protein components that can be transferred to target cells, thereby influencing their functions. Given that EVs serve as natural carriers of bioactive molecules, exhibit ease of cellular uptake, possess angiogenic properties, and demonstrate low immunogenicity, they hold significant promise in the context of cardiac regenerative therapy [300, 301], as is shown in Figure 4. Cardiac‐related exosomes are typically derived from high‐induction pluripotent stem cell‐derived cardiomyocytes (hiCM‐EV) or primitive high‐induction pluripotent stem cells (hiPSC‐EV). Research has demonstrated that exosomes originating from hiPSC‐CMs induce microvascular sprouting from aortic rings in vitro, maintain intracellular calcium homeostasis, and reduce apoptosis. More importantly, in vivo experiments, these exosomes improved cardiac function and enhanced heart regeneration indicators, such as angiogenesis and wall stress in infarcted regions, when compared with the MI group [302]. This acellular therapeutic approach also did not increase the incidence of arrhythmia complications. Wei et al. [303] discovered that endothelial‐derived EVs delivering circWhsc1 to cardiomyocytes can activate the TRIM59/STAT3 signaling pathway, thereby inducing cardiomyocyte proliferation and restoring cardiac structure and function in adult mice following MI. Furthermore, researchers have sought to enhance the delivery and targeting efficiency of exosomes through various modifications. For instance, the incorporation of modified glycerophospholipid–polyethylene glycol conjugates into the solution from which exosomes are isolated has been employed [304]. Another approach involves the genetic modification of the cells producing the exosomes, such as the addition of targeting peptides like peptide IMTP (CSTSMLKAC) fusion with Lamp‐2b targeting peptides on the surface of the vesicles to specifically target ischemic myocardial regions [305]. In addition to targeting capabilities, the retention time of EVs in the heart significantly influences their therapeutic efficacy. Wei et al. [306] overexpressed the transmembrane protein CD47 in MSCs, resulting in the modification of the EVs surface with CD47. The interaction between CD47 and the signaling protein α inhibits the phagocytic activity of mononuclear macrophages toward the EVs. The study found that the modified vesicles remained significantly enriched in the infarcted myocardium even 8 h postinjection, leading to improved cardiac function in mice. Currently, an emerging direction in exosome‐based therapies involves the combination of exosomes with biomaterials, such as injectable myocardial patches, to further enhance therapeutic efficacy. For instance, existing research has demonstrated that an injectable hydrogel patch can gradually release exosomes secreted by cardiomyocytes derived from hiPSCs, thereby facilitating sustained delivery of exosomes. When implanted into the infarcted hearts of rats, the hydrogel patch that releases exosomes has been shown to promote the recovery of left ventricular function, reduce the burden of arrhythmias, and decrease cardiomyocyte apoptosis within 24 h postinfarction [307]. Compared with traditional stem cell therapy, EVs exhibit superior natural biocompatibility, with lower risks of tumorigenicity and arrhythmia, reduced immune rejection, and no need to consider cell survival rates. Furthermore, in contrast to conventional drug or gene editing therapies, EVs enable multitarget regulation and, through engineered targeting modifications, can more effectively act on specific heart regeneration areas. By integrating various biomaterials, EVs can also achieve sustained release and synergistic multimechanism therapy.

However, the mechanisms by which components carried by naturally sourced EVs promote cardiac repair and regeneration remain unclear, necessitating further research for exploration. Additionally, there is a paucity of studies focused on optimizing the targeting and retention capabilities of EVs themselves. In terms of clinical application, the methods for isolating and purifying EVs vary widely, leading to considerable discrepancies in the purity, size, and concentration of the obtained vesicles [308]. Currently, the isolation and purification techniques for EVs are time‐consuming and yield relatively low quantities, particularly since cardiac repair typically requires high doses of EVs. Moreover, EVs cannot mechanically stabilize the thinning ventricular wall or provide the structural cues necessary for organized tissue reconstruction. In terms of safety and targeting, the modification process may introduce immunogenicity or interfere with vesicle functionality. Additionally, unintended complex biomolecules carried by the vesicles, may trigger ectopic fibrosis or systemic toxicity [309]. Long‐term safety data, particularly human follow‐up studies, remain critically lacking. Regarding clinical administration routes, there is no consensus on whether to administer EVs via intramyocardial injection or intravenous injection, nor is there a standardized protocol for optimal dosing and administration strategies. Therefore, these critical limitations underscore the complementary role of engineered cardiac patches, which are specifically designed to achieve mid‐to‐long‐term structural and functional cardiac recovery through physical support, guided cellular alignment, and the integration of mechanical and electrical signals.

#### 4.4.2 Engineered Tissue Patches for Heart Regeneration

The fabrication of cardiac patches is derived from two types of biomaterials: synthetic polymers and natural biomaterials. It is crucial for cardiac patches to incorporate a microstructure that physiologically mimics the natural heart, as this provides the necessary microenvironment for the implanted matrix and offers mechanical support to the structure itself [310], as is shown in Figure 4. Natural biomaterials exhibit superior biocompatibility and can more effectively replicate the ECM microenvironment, thereby facilitating in vivo biochemical signaling. Conversely, synthetic materials possess a high degree of customizability and modifiability. Consequently, hybrid scaffolds composed of both natural and synthetic materials have emerged as a focal point in recent advancements in heart regeneration and repair, as this approach capitalizes on the advantages offered by both categories of materials [311]. For instance, polycaprolactone/gelatin patches effectively limit ventricular dilation following MI and reduce adverse remodeling of the ventricular wall [312]. Most cardiac repair patches rely on the therapeutic components they contain to promote heart regeneration and angiogenesis in the infarcted area. These components may include cells or bioactive molecules, such as growth factors, exosomes, miRNAs, and even therapeutic drugs. In terms of cellular components, MSCs are widely utilized in cardiac repair materials due to their ease of procurement, low immunogenicity, and ability to secrete various immunomodulatory and tissue regeneration‐promoting factors. For example, the coinjection of hiPSC‐CMs and supportive cell types (endothelial cells and smooth muscle cells) in cardiac patches has demonstrated improved cell engraftment and enhancements in myocardial wall stress, apoptosis, vascularization, and contractile function in porcine and murine models of MI [313, 314]. Another study reported a novel patch developed by suspending iPSCs, such as CMs, SMCs, and ECs, within a fibrin scaffold. This myocardial patch demonstrated the ability to enhance cardiomyocyte proliferation in vitro for a duration of up to 7 days, while significantly reducing the area of damage and improving cardiac function in a MI pig model [315]. Regarding bioactive molecules, one study incorporated alginate microspheres into collagen‐based patches to provide controlled release of HGF and IGF‐1, thereby enhancing the migration and proliferation capabilities of endogenous cardiac stem cells [316]. However, traditional cardiac patches face a significant challenge: they can only remain on the epicardial surface, resulting in the restoration of only the superficial myocardium while offering limited repair efficacy for the deeper damaged myocardial layers. Recent research has developed a scaffold‐free patch loaded with MSCs based on ferumoxytol, which not only autonomously recognizes damage signals and migrates into the injured myocardium but also allows for noninvasive tracking via magnetic resonance imaging to assess the success of transplantation at an early stage, significantly enhancing the efficacy of cardiac repair [317]. In contrast to conventional therapies that merely improve cardiomyocyte function or delay structural remodeling, cardiac patches can directly provide mechanical support and guide the directional migration, regeneration, and differentiation of cardiomyocytes, holding promise for achieving complete structural and functional restoration. Moreover, a single implantation offers prolonged mechanical stability, thereby circumventing the long‐term toxicity associated with pharmacological interventions. Additionally, cardiac patches can reconstruct electrical conduction in the infarcted zone through biomimetic structures, restoring partial contractile function of cardiomyocytes—a feat unattainable by traditional drugs or stem cell transplantation [318].

Cardiac tissue patches also face challenges for clinical application. First, the current implementation of cardiac patch constructs requires thoracotomy. Many patients are too frail to withstand the trauma associated with such invasive procedures, necessitating the exploration of novel implantation methods. Additionally, scaffold material properties must be personalized to maintain high biocompatibility and degradability while preserving their capacity to support cardiac regeneration. Second, the vascularization structure and tissue architecture of cardiac patches need to be optimized to achieve faster and more mature vascular development postimplantation. Various strategies have been employed to achieve platform pre‐vascularization or develop adaptive cardiac patches [319]. Further development of highly vascularized adaptive patches that better mimic native myocardial tissue may yield more clinically significant outcomes. Third, consideration must be given to whether the patch can fully integrate with the host heart's electrical signals to effectively promote intercellular electrical conduction and synchronized contraction, thereby improving cardiomyocyte functionality. Finally, for cellular patches, the efficiency of obtaining sufficient therapeutic cells and the maturity of the hiPSC‐CMs used must be addressed. With recent advances in cell engineering to obtain more mature hiPSC‐derived tissues, combinatorial strategies may enable the application of autologous, patient‐specific cardiac patches, minimizing rejection risks. To enhance the integration and therapeutic efficacy of cardiac patches, and to overcome the above limitations, injectable biomaterial platforms, particularly hydrogels, emerge as key enablers.

#### 4.4.3 Hydrogels and Hydrogel Derivatives for Heart Regeneration

Hydrogels are a class of three‐dimensional network structures formed by hydrophilic polymers through chemical or physical cross‐linking, capable of rapidly swelling in water while retaining a significant amount of water without dissolving [320]. Hydrogels exhibit excellent biocompatibility, biodegradability, and environmental responsiveness, making them widely applicable in the field of biomedical engineering, particularly in cardiac tissue engineering [321, 322, 323]. They can serve as a synergistic strategy for myocardial repair following MI by encapsulating various bioactive substances, such as growth factors, stem cells, and nucleic acids, to promote heart regeneration, vascularization, and functional recovery [324]. Additionally, hydrogels can be utilized as injectable minimally invasive technologies, overcoming the limitations of traditional scaffold techniques by modulating swelling behavior and localized delivery, thereby enhancing therapeutic efficacy and patient compliance [325].

Hydrogel‐based myocardial patches can be implanted surgically or injected directly to cover or fill the infarcted area, providing mechanical support, preventing ventricular remodeling, increasing cardiac wall thickness, and improving cardiac function. Hydrogels can also serve as carriers for drugs, growth factors, matrix molecules, and stem cells, facilitating sustained or controlled release to promote the regeneration and repair of myocardial tissue. For instance, a cross‐linked herbal macromolecular hydrogel has been developed to deliver rat bone marrow MSCs (rBMSCs) @ microgels for the treatment of MI. This hydrogel exhibits excellent biocompatibility and mechanical properties, effectively protecting rBMSCs from ischemic and oxidative stress‐induced damage, while simultaneously enhancing the efficacy of their secreted factors to promote angiogenesis and cardiac function recovery [326]. At the same time, the hydrogel patch needs to re‐establish coupling with the electrical activity of the host heart and improve electrophysiological function, which is crucial for preventing asynchronous contractions of the ventricular cavity and malignant arrhythmias. A novel self‐adhesive hydrogel patch is composed of conductive polydopamine hybridized PEDOT nanoparticles (PPEDOTNPs) and polygallic acid–interpenetrating gelatin methacrylamide networks. Due to their hydrophilicity, PPEDOTNPs are uniformly distributed throughout the polymer network, establishing well‐connected electrical pathways that exhibit stable conductivity. This hydrogel, characterized by its intrinsic conductivity, is crucial for the maintenance of electrical conduction following MI [327].

Hydrogels can also be injected into the heart chamber or pericardial space in a liquid or semi‐solid form through minimally invasive techniques, which form one or more layers of hydrogel that serve to protect and regulate cardiac function. Depending on various stimulus conditions, hydrogels can undergo changes such as crosslinking, solidification, and degradation in vivo, thereby rendering them as stimulus‐responsive hydrogels capable of responding to and modulating the microenvironment of MI [328]. One of the key roles of injectable hydrogels is to promote angiogenesis, which is critical for heart regeneration. Research has demonstrated that the injection of hydrogels into the epicardial myocardium can enhance neovascularization and alleviate adverse pathological remodeling [329]. Due to their mechanical properties, hydrogels can maintain perfusable vascular structures while providing sufficient hydraulic support for blood flow. Furthermore, they offer entrance for vessels and promote in situ vascularization through their interconnected porosity. Injectable hydrogels can also enhance the geometric configuration of the left ventricle to provide support for myocardial tissue and promote the regeneration of myocardial tissue [330]. Concurrently, injectable hydrogels can alleviate the pressure on the ventricular wall, thereby mitigating the extent of infarction and further dilation of the left ventricle [331]. Another significant application of injectable hydrogels is their integration with cellular or drug therapies. For example, an injectable hydrogel composed of PLGA nanoparticles encapsulating curcumin (PLGA@CurNPs) and recombinant human type III collagen can effectively reduce the levels of ROS and cellular apoptosis following MI, while simultaneously promoting cell proliferation, migration, and angiogenesis [332]. Injectable hydrogels have also entered numerous clinical trials, including an exciting global first study on transcatheter endocardial injection of implantable alginate hydrogel, which has completed its first‐in‐man (FIM) study (*n* = 12) to explore safety and feasibility. The results indicate that alginate hydrogel demonstrates a favorable therapeutic effect for patients with heart failure. Notably, the 180‐day postoperative follow‐up results show improvements in both left ventricular EDV (LVEDV) and left ventricular ESV (LVESV), providing evidence that left ventricular dilation and remodeling have been reversed [333]. In conclusion, compared with traditional drug therapies, hydrogels, as effective carriers of bioactive molecules, can achieve minimally invasive delivery and controlled release in vivo. Hydrogels can also effectively address the problem of low inhibition rate of traditional stem cells. The survival rate of encapsulated stem cells is greatly improved. Compared with traditional biomaterials, hydrogels, with their high binding affinity, swelling and adsorption capacities, and colonization resistance, make it easier for researchers to utilize and effectively develop them to suit the special characteristics of the heart, such as its rapid and continuous contraction, high conductivity, and rich blood circulation.

However, as a soft material, hydrogels typically exhibit an elastic modulus lower than that of myocardial tissue. This mechanical mismatch may interfere with normal cardiac contraction and relaxation functions. Consequently, it is necessary to design and develop hydrogels with both high elastic modulus and high ductility to accommodate the high‐strain and high‐stress environment of the heart. Additionally, since hydrogels are nonconductive materials, their impedance mismatch with myocardial tissue may disrupt the propagation of cardiac electrophysiological signals, potentially leading to arrhythmias or cardiac arrest. Therefore, conductive hydrogels must be engineered to achieve electromechanical coupling with myocardial tissue. Furthermore, most current hydrogel studies rely on animal experiments, and their immunogenic potential remains incompletely elucidated, particularly in vivo. Significant improvements in hydrogel performance are still required for clinical translation. Although hydrogel implantation techniques have substantially reduced injury risks, minimally invasive approaches—especially for hydrogel‐based cardiac patches—require further development. For patients with mild‐to‐moderate heart failure, open‐chest surgery is not a viable option. In the future, minimally invasive interventional techniques and reliable delivery systems (e.g., injectable hydrogels, coatable patches, and suture‐free adhesive patches) may offer novel solutions. Last, bioactive hydrogels, which are currently prevalent in research, must also account for factors such as drug release kinetics, drug stability, dosage, and synergistic effects to ensure therapeutic efficacy in vivo. Nevertheless, it is undeniable that hydrogels, as a biomaterial with multiple advantages and considerable potential, hold broad prospects for application in heart regeneration and repair (Figure 4). Beyond the delivery of cells, drugs, and genetic material facilitated by EVs, patches, and hydrogels, modulating the cardiac microenvironment and cellular activity through noninvasive physical stimuli such as light represents another frontier.

#### 4.4.4 PBM Regulation for Heart Regeneration

PBM refers to nonthermal photons with relatively low power (1–500 mW) and spectra within the visible or near‐infrared range (400–1000 nm), which can trigger either activating or inhibitory biological responses in cells and tissues [334]. This bidirectional regulatory PBM encompasses both the previously dominant low‐level laser therapy (LLLT) and the more functionally advantageous light‐emitting diode therapy. PBM primarily elicits physical responses in organisms through stimulation by low‐power light beams of specific wavelengths [335, 336], typically restoring pathological states to normal levels by modulating the immune system, nervous system, circulatory system, and various tissue metabolic systems, thereby achieving therapeutic effects. More importantly, a growing body of experimental evidence demonstrates that the combined application of PBM with conventional cardiac interventions yields superior outcomes in repairing and ameliorating MI‐related diseases [337]. Specifically, PBM can improve cardiac structure following MI. Yaakobi et al. [338] early demonstrated the positive effects of PBM in reducing infarct size in rats. Through photostimulation experiments conducted after ligating the left anterior descending coronary artery in rat hearts, it was found that low‐power‐density laser irradiation significantly decreased the extent of MI. As the duration of MI increased, the reduction rate of infarct area exhibited a progressive upward trend. Furthermore, the left ventricular dilation volume correspondingly decreased, indicating that LLLT possesses sustained therapeutic characteristics in repairing MI tissue. The study further confirmed that PBM could modulate VEGF and promote angiogenesis in the peri‐infarct zone. Red and near‐infrared light are the primary light sources for PBM. Numerous studies have demonstrated that red light possesses the capability to promote the proliferation of neural stem cells and skeletal muscle satellite cells [339, 340]. However, research on whether PBM exerts direct effects on cardiomyocyte proliferation and heart regeneration remains remarkably limited. Gao et al. [341] first demonstrated that 630 nm LED red light irradiation suppresses the expression of miR‐136‐5p in cardiomyocytes, thereby relieving its inhibitory effect on the INO80 gene and subsequently promoting G1/S phase transition and cell division in cardiomyocytes. Additionally, red light can simultaneously enhance the pentose phosphate pathway to provide biosynthetic precursors for proliferation. Subsequent studies developed upconversion cyanobacteria nanocapsules coated with light‐responsive hydrogels (UCCy@Gel), which utilize upconversion nanoparticles to convert near‐infrared light (980 nm) into visible light, thereby activating cyanobacterial photosynthesis for oxygen release. During the prevention phase under dark conditions, cyanobacterial respiration consumes oxygen, inducing the expression of heat shock protein 70 (HSP70) in cardiomyocytes and reducing apoptosis. In the subsequent treatment phase, near‐infrared irradiation triggers photosynthesis, releasing oxygen to suppress macrophage M1 polarization, decrease proinflammatory cytokine levels (e.g., IL‐6, TNF‐α), and promote angiogenesis [342]. Although no direct evidence of cardiomyocyte proliferation was observed, these findings indirectly highlight the significant potential of PBM in creating a favorable microenvironment for heart regeneration.

It should be clarified that research on PBM for heart regeneration represents an emerging interdisciplinary field spanning multiple disciplines and levels, involving comprehensive experimental studies integrating optics, mechanics, physiology, and biochemistry. Currently, LED light sources offer distinct material advantages, including low cost, portability, ease of operation, low energy consumption, and long diode lifespan. Compared with lasers, they provide broader radiation coverage and enhanced safety [343]. Notably, red light therapy, characterized by its high wavelength selectivity, strong controllability, and absence of thermal effects, warrants in‐depth exploration in future regenerative applications. However, most studies on PBM remain confined to animal experimentation. Given the physiological structural differences between humans and animal models such as mice and canines, careful consideration must be given to the energy penetration, absorption characteristics, and dosage selection of light sources in both animal and human tissues. The research teams of Gepstein and Kornowski proposed delivering optical fiber catheters to human myocardium using nonfluorescent in vivo navigation and positioning technology, while also developing fiber‐optic transmission techniques for laser delivery and percutaneous pretreatment protocols for LLLT on myocardial surfaces [344, 345]. Furthermore, numerous questions regarding the mechanistic underpinnings of how PBM influences heart regeneration remain to be elucidated.

### 4.5 Preclinical Animal Experiments and Clinical Trials

Preclinical models, spanning rodents, sheep, and nonhuman primates, have provided critical insights into the mechanisms of cardiomyocyte proliferation, tissue remodeling, and functional recovery postinjury. For instance, studies in fetal sheep demonstrated inherent resistance to ischemic damage, with minimal inflammatory activation and preserved metabolic pathways, contrasting sharply with adolescent models where dysregulated inflammation and adverse remodeling predominated [346]. Similarly, engineered approaches using CCND2‐modified mRNA or tissue‐engineered heart muscle (EHM) allografts in rodents and pigs showed that targeted CM cell‐cycle activation and epicardial grafting improve left ventricular function, reduce infarct size, and promote vascularization without arrhythmogenic risks [347, 348]. These findings underscore the importance of species‐specific responses and the role of developmental‐stage‐dependent regenerative capacity (Table 6).

**TABLE 6:** Preclinical animal experiments and clinical trials.

Species	Treatment	Mechanism	References
Pig	AAV9–Sav–shRNA induce endogenous cardiomyocyte renewal after myocardial infarction in adult pig	Inducing Hippo pathway deficiency	[342]
	The CCND2‐cardiomyocyte‐specific modified mRNA translation system promoted cardiomyocyte proliferation in pig with MI	Facilitating DNA synthesis and cell cycle progression	[345]
Sheep	A sheep model of MI induced by ligating the left anterior descending coronary artery performed on fetuses (105 days) and adolescent sheep (6 months)	Hippo and Meis cell proliferation pathway	[347]
		Glucose and fatty acid regulation	[351]
		Neuregulin signaling	
Human	A newborn had acute MI due to left coronary artery thrombosis, and after thrombolytic therapy, the LV myocardium recovered with function nearly normal.	Thrombolytic therapy with r‐tPA	[352]

Translating these insights to clinical settings, early‐phase trials have explored safety and feasibility. A randomized trial, using collagen scaffolds with mesenchymal stromal cells, established safety but highlighted limited efficacy, emphasizing the need for larger trials to assess therapeutic potential. Case reports and first‐in‐human EHM implantation provided proof‐of‐concept for acute and chronic myocardial repair, demonstrating retained graft viability, functional vascularization, and improved contractility [348]. Notably, clinical data revealed challenges such as immune responses to allografts and the necessity for optimized immunosuppression to balance efficacy and safety.

Key gaps remain, including long‐term engraftment stability, scalability of cell‐based therapies, and mitigation of arrhythmic or fibrotic complications. The heterogeneity in preclinical outcomes—for example, osteochondral differentiation in primate EHM grafts—calls for refined differentiation protocols [348]. Furthermore, combining biomaterial scaffolds with genetic or cellular interventions may synergize CM proliferation and tissue integration [118]. Future studies should prioritize large‐animal models that better mimic human pathophysiology and incorporate multimodal imaging validate mechanistic outcomes. Collectively, these efforts bridge preclinical innovation with clinical translation, advancing toward therapies that address the unmet need for heart regeneration in heart failure.

## 5 Conclusion and Perspectives

In summary, this article reviews the maturation process of cardiomyocytes using the example of postnatal maturation of mammalian cells, elucidating various pathophysiological mechanisms associated with heart regeneration. Beyond the currently prevalent single‐cell transcriptomics, future research will necessitate the application of additional omics approaches such as spatial transcriptomics, spatial proteomics, and epigenomics to further elucidate the molecular mechanisms underlying cardiomyocyte maturation [349].

More importantly, based on the aforementioned findings, several intervention targets for basic research and therapeutic strategies entering clinical trials have been identified. However, over the past decade, studies on the exogenous transplantation of stem cells have shown limited efficacy in enhancing heart regeneration, primarily functioning through paracrine effects. Therefore, current research on heart regeneration should focus on promoting endogenous adult cardiac myocyte proliferation to facilitate heart regeneration, particularly through the induction of metabolic reprogramming in cardiomyocytes. The advent of CRISPR–Cas9 and base‐editing technologies offers unprecedented opportunities to reactivate dormant regenerative programs in adult cardiomyocytes. For example, targeted of key enzymes for glucose metabolism could bypass cell cycle barriers to regeneration [350]. Concurrently, synthetic biology approaches—such as engineered macrophages secreting proregenerative factors OSM, VEGF—could create localized niches to support cardiomyocyte division. However, challenges persist, including off‐target effects, delivery efficiency, and immune rejection of edited cells. Developing cardiac‐specific viral vectors or lipid nanoparticles with tropism for cardiomyocytes may enhance precision. Additionally, combining gene editing with biomaterials could enable spatiotemporal control of therapeutic interventions.

AI is poised to revolutionize cardiac regenerative medicine. With the advancement of AI, machine learning methods can widely correct dysregulated gene networks in induced iPSCs, aiding in the development of novel therapeutics [351]. AI‐driven molecular docking may also optimize the design of fusion proteins to enhance receptor specificity. Beyond pharmacology, AI can guide the development of 3D‐bioprinted patient‐specific vascular architectures or predict the biomechanical properties of hydrogels tailored to infarcted regions [352]. Moreover, AI's role extends beyond the mere architectural design of cardiac patches. It can also predict the biomechanical properties of these bioprinted constructs, ensuring that they not only fit anatomically but also functionally integrate with the patient's existing tissues. The integration of AI with 3D bioprinting technologies has opened new avenues for creating functional cardiac tissues that exhibit contractility, conductivity, and vascularization, which are essential for successful cardiac repair and regeneration [353]. However, ethical concerns, such as data privacy in personalized genomics and algorithmic bias in model training, must be addressed to ensure equitable clinical translation.

Overall, in order to further advance the research and translational application of myocardial cell regeneration therapy, we face a series of critical scientific and translational issues that remain to be addressed. First, the study of myocardial cell regeneration requires more precise detection tools. As previously mentioned, due to the characteristic of entering the cell cycle without undergoing cytokinesis, the current detection metrics or tools do not fully and accurately reflect the proliferation levels of cardiomyocytes. Whether it is through assessing the cell cycle or indirectly reflecting myocardial cell proliferation based on the pairing or colony size of actively cycling or utilizing genetic engineering techniques to label proliferating cardiomyocytes, there exists a challenge of either overestimating or underestimating the proliferative capacity of cardiomyocytes. Second, myocardial cell proliferation is a continuous process that encompasses dedifferentiation, proliferation, and redifferentiation. Although current research has provided a relatively comprehensive understanding of the proliferation phase, studies on dedifferentiation and redifferentiation remain comparatively limited. Therefore, future research should place greater emphasis on exploring the stages of dedifferentiation and redifferentiation in myocardial cells. Moreover, effectively controlling the extent and localization of cardiomyocyte proliferation is crucial for translational applications, as excessively active myocardial cell proliferation may lead to the development of cardiac tumors [260]. Finally, drug and target screening necessitate models that closely resemble the human heart and possess higher maturity to facilitate clinical translation. Currently, the primary models for gene and drug screening include zebrafish embryos, neonatal mouse and rat cardiomyocytes, transgenic mouse cardiomyocytes, as well as cardiomyocytes, derived from ESCs or hiPSCs. The introduction of high‐throughput, mature cardiac organoid gene and drug screening platforms will play a pivotal role in promoting heart regeneration.

## Author Contributions

Mingchuan Liu, Tingwei Peng, and Rui Yu were responsible for completing the first draft. Kexin Wang, Di Wang, and Xiaojie Jia made substantial contributions to revising the first draft. Yan Zhang and Jianqiang Hu were responsible for the creation of figures and tables, as well as the provision of figure legends. Bingchao Qi and Yan Li were the corresponding authors. They were jointly responsible for designing the overall writing framework of the manuscript and reviewing and revising the entire manuscript to ensure that it meets high academic standards. All authors have read and approved the final manuscript.

## Ethics Statement

The authors have nothing to report.

## Conflicts of Interest

The authors declare no conflicts of interest.

## Data Availability

All data generated or analyzed during this study are included in this published article
